# Reversible and irreversible inhibitors of coronavirus Nsp15 endoribonuclease

**DOI:** 10.1016/j.jbc.2023.105341

**Published:** 2023-10-11

**Authors:** Jerry Chen, Rabih Abou Farraj, Daniel Limonta, Seyed Amir Tabatabaei Dakhili, Evan M. Kerek, Ashim Bhattacharya, Filip M. Reformat, Ola M. Mabrouk, Benjamin Brigant, Tom A. Pfeifer, Mark T. McDermott, John R. Ussher, Tom C. Hobman, J.N. Mark Glover, Basil P. Hubbard

**Affiliations:** 1 Department of Pharmacology, University of Alberta, Edmonton, Alberta, Canada; 2 Department of Biochemistry, University of Alberta, Edmonton, Alberta, Canada; 3 Department of Cell Biology, Li Ka Shing Institute of Virology, University of Alberta, Edmonton, Alberta, Canada; 4 Quantitative Biosciences Institute (QBI), University of California San Francisco, San Francisco, California, USA; 5 Gladstone Institute of Data Science and Biotechnology, Gladstone Institutes, San Francisco, California, USA; 6 Faculty of Pharmacy and Pharmaceutical Sciences, University of Alberta, Edmonton, Alberta, Canada; 7 Department of Pharmacology and Toxicology, University of Toronto, Toronto, Ontario, Canada; 8 Department of Chemistry, University of Alberta, Edmonton, Alberta, Canada; 9 Department of Clinical and Molecular Medicine, Norwegian University of Science and Technology, Trondheim, Norway; 10 High Throughput Biology Facility, Life Sciences Institute, University of British Columbia, Vancouver, British Columbia, Canada

**Keywords:** SARS-CoV-2, COVID-19, immune evasion, coronaviruses, nidoviruses, RNA endonucleases, Nsp15, high-throughput chemical screen, small-molecule inhibitors, COVID-19 therapeutics, hexachlorophene, IPA-3

## Abstract

The emergence of severe acute respiratory syndrome coronavirus 2, the causative agent of coronavirus disease 2019, has resulted in the largest pandemic in recent history. Current therapeutic strategies to mitigate this disease have focused on the development of vaccines and on drugs that inhibit the viral 3CL protease or RNA-dependent RNA polymerase enzymes. A less-explored and potentially complementary drug target is Nsp15, a uracil-specific RNA endonuclease that shields coronaviruses and other nidoviruses from mammalian innate immune defenses. Here, we perform a high-throughput screen of over 100,000 small molecules to identify Nsp15 inhibitors. We characterize the potency, mechanism, selectivity, and predicted binding mode of five lead compounds. We show that one of these, IPA-3, is an irreversible inhibitor that might act *via* covalent modification of Cys residues within Nsp15. Moreover, we demonstrate that three of these inhibitors (hexachlorophene, IPA-3, and CID5675221) block severe acute respiratory syndrome coronavirus 2 replication in cells at subtoxic doses. This study provides a pipeline for the identification of Nsp15 inhibitors and pinpoints lead compounds for further development against coronavirus disease 2019 and related coronavirus infections.

Since its appearance in late 2019, severe acute respiratory syndrome coronavirus 2 (SARS-CoV-2) has infected hundreds of millions of individuals globally and killed more than 18 million people (1, 2). Exposure to this virus can lead to respiratory problems, systemic inflammation, and multiorgan dysfunction, which collectively define coronavirus disease 2019 (COVID-19) (1). Coronaviruses are characterized by large single-stranded positive sense RNA genomes of roughly 30 kB and a “corona” structure in their envelope (3, 4, 5). SARS-CoV-2 is classified as a lineage B betacoronavirus alongside its most phylogenetically similar relative, SARS-CoV-1 (4). Translation of the SARS-CoV-2 viral genome is performed by host cell ribosomes and generates four structural proteins: an envelope protein involved in viral assembly and budding, a membrane protein involved in defining virion shape, a nucleocapsid protein that packages genomic RNA, and a spike protein involved in host attachment and entry (6, 7). SARS-CoV-2 also contains 16 nonstructural proteins (Nsps) including the 3C-Like main protease (3CLpro/MPro, Nsp5), the papain-like protease (PLPro, Nsp3), and the RNA-dependent RNA polymerase (RdRp) complex (Nsp7, Nsp8, and Nsp12) (5, 8, 9). Finally, nine open reading frames that encode additional accessory proteins have been annotated (5, 8, 9).

Current strategies to combat COVID-19 have focused on the development of prophylactic vaccines mostly directed against the SARS-CoV-2 spike protein (10) and small-molecule drugs targeting the 3CLpro (11), PLPro (12), and RdRp complex (13). Recently, the Food and Drug Administration approved the first two small-molecule drugs, nirmatrelvir/ritonavir and molnupiravir, which target the 3CLpro (14) and RdRp (15) complex, respectively, for therapeutic use. Despite these advances, transmission of SARS-CoV-2 continues in the population, and severe outcomes including death persist (16). This is in part because of the evolution of new viral variants that acquire mutations in genes that encode vaccine-induced antibody targets (16, 17). Furthermore, it has been suggested that current pharmaceutical treatments against COVID-19 could also be rendered ineffective in the future because of resistance (18). Thus, there is a need to broaden the repertoire of coronavirus antiviral treatments to additional targets in the SARS-CoV-2 proteome.

A promising but less explored SARS-CoV-2 drug target is Nsp15, a nidoviral endoribonuclease that selectively cleaves 3′ of uridylates (NendoU) to generate 2′,3′-cyclic phosphodiester and 5′-hydroxyl termini products (19, 20). Nsp15 cleaves both ssRNA and dsRNA but not DNA (20, 21). Structurally, it is comprised of a hexamer formed by the dimerization of two trimers (20). X-ray crystal (20) and cryo-EM structures (22) reveal that each monomeric unit has three domains: an N-terminal domain involved in multimerization, a middle domain, and the C-terminal catalytic core (20). The active site contains residues that are reminiscent of the catalytic triad found in RNase A, including two histidine residues (His235 and His250) that act as general acid–base catalysts and a lysine residue (Lys290) (19, 20). Two other important residues within the active site are Ser294 and Tyr343, which are thought to enforce uracil specificity (20). Unlike RNase A, Nsp15 requires coordinated manganese ions (Mn^2+^) for catalysis (20). These ions are purported to maintain active site conformation and substrate binding (20).

The role of Nsp15 in coronavirus replication and pathogenesis is complex and multifaceted (5). Several studies have suggested an essential role for Nsp15 in viral replication, as mutation of Nsp15 blocks viral RNA synthesis in certain cell types (23) and attenuates disease phenotypes in mice (24). Experimental data suggest that Nsp15 also plays an important role in shielding viruses from innate host cell immunity (24). It has been proposed that Nsp15 facilitates evasion of dsRNA sensors that would normally activate a type I interferon (IFN) response by cleaving viral dsRNA outside the replication complexes (24). This is supported by studies with SARS-CoV-1 showing that Nsp15-defective viruses induce MDA5, PKR, and OAS/RNase L dsRNA sensing pathways (5, 24, 25, 26) and by studies with SARS-CoV-2 implicating Nsp15 in suppression of IFNβ production (27). Finally, recent work has shown that Nsp15 cleaves 5′ polyuridines from negative sense viral RNA (PUN RNA) and thereby decreases MDA5-mediated IFN response (28). While its multiple roles in viral biology are still being elucidated, it is apparent that Nsp15 plays a critical role in coronavirus pathogenesis (24) and is therefore an attractive candidate for drug design.

Only a few Nsp15 inhibitors have been experimentally validated to date (5). These include benzopurpurin B, which inhibits RNase A and numerous nidoviral Nsp15 homologs (19), betulonic acid derivatives that inhibit Nsp15 from HCoV-229E but not SARS-CoV-2 viruses (29), and tipiracil, which acts as a competitive inhibitor of Nsp15 (30). The β-amyloid antiaggregation molecule Exebryl-1 also robustly inhibits SARS-CoV-2 Nsp15 *in vitro* but displays weaker potency in cells (EC_50_ of roughly 65 μM in Calu-3 cells) (5). While these and other studies (31, 32) have clearly demonstrated the potential to chemically inhibit Nsp15, small-molecule scaffolds that are potent, bioactive, and nontoxic remain elusive. Here, we perform a high-throughput screen (HTS) of over 100,000 diverse compounds to identify Nsp15 inhibitors that meet these criteria. We refine our initial list of hits by using a workflow that encompasses a series of biochemical and biophysical experiments to eliminate pan-assay interference (PAIN) compounds (33). We then characterize the potency, selectivity against other Nsp15 orthologs, kinetic mechanisms, predicted binding mode, and cellular activity/toxicity of five lead compounds. These include the antimicrobial drug hexachlorophene (34), the PAK1 kinase inhibitor IPA-3 (35), and three proprietary molecules (CID5220994, CID5266986, and CID5675221) from the ChemBridge DIVERSet library. We find that these inhibitors operate through reversible mechanisms, with the exception of IPA-3, which is irreversible and likely covalent. Moreover, we demonstrate that hexachlorophene, IPA-3, and CID5675221 inhibit SARS-CoV-2 replication in Vero CCL-81 cells at subtoxic doses. Overall, this work provides a comprehensive platform for the identification and validation of small-molecule Nsp15 inhibitors and identifies several potent, bioactive, and relatively nontoxic lead compounds with potential for further development into treatments against COVID-19 and related illnesses.

## Results

### Measurement of SARS-CoV-2 Nsp15 enzyme kinetics

**Figure 1:**
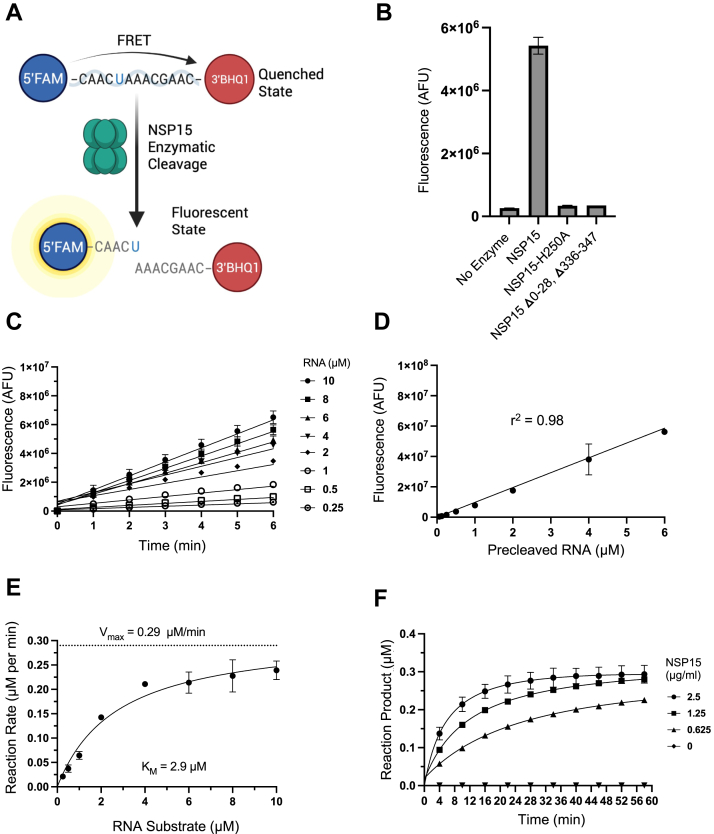
**Characterization of Nsp15 activity using an FRET-based assay.***A*, schematic of Nsp15 activity assay. An RNA substrate flanked by 5′fluorescein (FAM) and 3′black hole quencher 1 (BHQ1) modifications is cleaved by Nsp15 in the presence of manganese. *B*, bar graph showing the activity of various Nsp15 mutant proteins using the FRET-based assay. Reactions were allowed to proceed for 40 min at 37 °C; mean ± SD is shown (n = 3). *C*, reaction progress over time at various concentrations of RNA substrate; mean ± SD shown (n = 3). Points were fit to linear equations. *D*, standard curve (linear fit) showing the relationship between RNA cleavage (using a positive control RNA lacking the quencher) and fluorescence; mean ± SD is shown (n = 3). *E*, plot showing the relationship between reaction rate and substrate concentration generated using data from *C*. *K*_*M*_ and *V*_max_ values were determined by fitting data to the Michaelis–Menten equation (v = *V*_max_[S]/(*K*_*M*_ + [S])) using GraphPad Prism; mean ± SD is shown (n = 3). *F*, reaction progress curves using a fixed concentration of 0.5 μM RNA at different concentrations of Nsp15 enzyme; mean ± SD is shown (n = 3). All experiments were repeated three times with similar results. Nsp, nonstructural protein.

To measure Nsp15 enzyme kinetics and determine the ideal parameters for a high-throughput compound screen, we adapted a previously described biochemical assay (21). This assay employs a monouridylated ssRNA substrate that is flanked by a 6-carboxyfluorescein (6-FAM) moiety at the 5′ end and a tetramethylrhodamine quencher at the 3′ end (21). Cleavage of the substrate by Nsp15 eliminates FRET between the fluorophore and quencher, resulting in a fluorescent signal (21). For our studies, we substituted the tetramethylrhodamine group with a 3′ black hole quencher (Fig. 1*A*), which has been demonstrated to yield more consistent results in biochemical assays (36). We purified recombinant SARS-CoV-2 Nsp15 from *Escherichia coli* and tested its ability to cleave several ssRNAs of different sequence length and composition alongside positive and negative controls (Fig. S1, *A* and *B*). We identified one sequence, RNA2 (5′-FAM-CAACUAAACGAAC-BHQ1-3′), which yielded low background and robust signal in the presence of Nsp15 (Fig. S1*B*). This substrate was used in subsequent experiments. Next, we verified that the signal being measured in the assay required Nsp15 activity using a catalytic histidine mutant (H250A) and an inactive monomeric truncated protein variant (Fig. 1*B*) (26, 37). In addition, we confirmed that the signal was enhanced by manganese ions (Fig. S1*C*) and abrogated by metal chelation with EDTA (Fig. S1*D*). These experiments validated the ability of our assay to reliably measure Nsp15 activity.

Enzyme and substrate concentrations and reaction time must be carefully chosen to ensure optimal hit identification when performing HTSs (38). To determine the *K*_*m*_ of RNA2, we first measured fluorescence over a time window for reactions containing variable amounts of substrate at a fixed enzyme concentration (Fig. 1*C*). In addition, we serially diluted precleaved (fluorescent) RNA to generate a standard curve relating fluorescence signal to the amount of product produced (Fig. 1*D*). Next, we plotted the slopes (derivatives) of the lines in Figure 1*C* *versus* RNA substrate concentration and used the standard curve equation (Fig. 1*D*) to transform the *y*-axis to reaction rate (Fig. 1*E*). Fitting this plot to a Michaelis–Menten curve yielded a *K*_*m*_ value of ∼3 μM for RNA2 and a *V*_max_ value of ∼0.3 μM/min, which are in agreement with recently published results (31). Finally, to optimize the enzyme concentration and time for our HTS, we generated reaction progress curves at a fixed substrate concentration (0.5 μM) at various enzyme concentrations. As shown in Figure 1*F*, we found that product cleavage progressed until saturation was reached between ∼30 and 60 min, depending on the enzyme concentration used. These data were used to guide the selection of parameters for our fixed end-point high-throughput compound screen.

### HTS for small-molecule inhibitors of SARS-CoV-2 Nsp15

**Figure 2:**
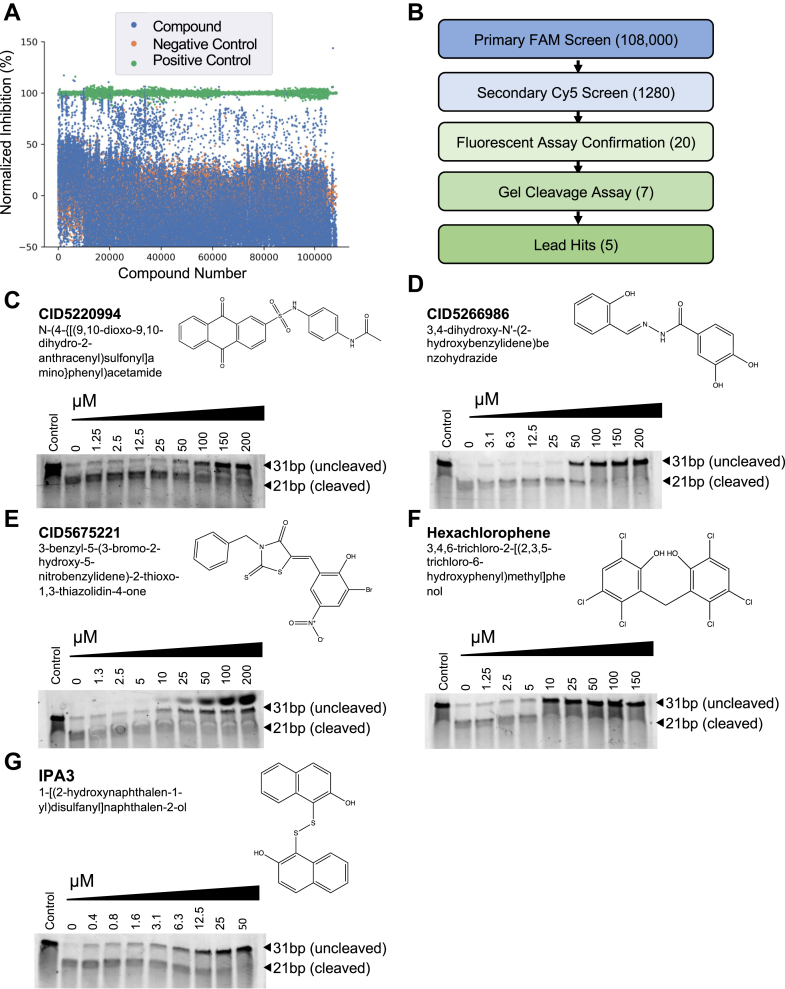
**Identification and validation of SARS-CoV-2 Nsp15 inhibitors.***A*, plot summarizing the results of the primary ∼108,000 compound high-throughput screen using the FAM-BHQ1 RNA substrate. DMSO was used as a negative control, and reactions without enzyme, or with enzyme in the presence of benzopurpurin B, were used as positive controls. *B*, schematic outlining the pipeline used to prioritize hits. Gels showing the results of Nsp15 native RNA cleavage assays in the presence of increasing concentrations of (*C*) CID5220994, (*D*) CID5266986, (*E*) CID5675221, (*F*) hexachlorophene, or (*G*) IPA-3. Nsp15 (7.5 ng/μl) and RNA substrate (25 ng/μl) were incubated with compounds and a 31-nt single “rU”-containing RNA substrate for 1 h at 37 °C. Bands representing the uncleaved substrate and the 21-nt cleaved product were visualized by performing denaturing gel electrophoresis followed by SYBR Gold staining. Representative gels are shown; experiments were repeated three times with similar results. BHQ1, black hole quencher 1; DMSO, dimethyl sulfoxide; FAM, carboxyfluorescein; Nsp, nonstructural protein; SARS-CoV-2, severe acute respiratory syndrome coronavirus 2.

The identification of Nsp15 inhibitors was accomplished by carrying out a screen of over 108,000 small molecules, comprised of compounds sourced from Maybridge, Prestwick, Microsource Spectrum, LOPAC-1280, TimTec, and ChemBridge DIVERSet collections. Overlap between compound libraries was <0.1%. After acoustic dispensing of buffer, enzyme, and compound into wells of 384-well plates, an initial read was performed to measure background and account for autofluorescence. Next, substrate was added, and reactions were allowed to proceed for ∼20 min before being stopped *via* the addition of 100 mM EDTA, at which time a second measurement was recorded. Finally, a 5′FAM RNA lacking a quencher was added to each reaction, and a third measurement was performed to account for potential signal quenching caused by each compound. Reactions with benzopurpurin B (inhibitor) and reactions without enzyme were used as positive controls on each plate (19), whereas reactions in the presence of 0.2% dimethyl sulfoxide (DMSO) were used as a negative control (no inhibition). The signal/noise ratio of accepted plates was >5 with an average Z′ score of 0.47 ± 0.11 (standard deviation). As this was a challenging assay with respect to read timing and reagent additions, plates with a Z′ score >0.3 were accepted as complete since the high signal/noise ratio enabled reasonably good distinction of actives. Plots summarizing the results of the screen are shown in Figure 2*A* and Fig. S2*A*. After filtering out compounds displaying high autofluorescence (read 1) or quenching (read 3–read 2), we identified 1280 reactions with fluorescence values >2 standard deviations from the mean. The corresponding compounds from these reactions were selected for further inspection, as outlined in the workflow in Figure 2*B*.

We performed a secondary screen of our initial hits using a substrate in which 5′FAM was replaced with 5′Cy5 (a red-shifted dye) to eliminate compounds yielding artifactual inhibition because of interaction with the 5′FAM group. The results of this experiment are shown in Fig. S2, *B* and *C*. Reactions without enzyme and reactions with enzyme and DMSO only were used as positive and negative controls, respectively. The 20 most potent inhibitors identified in the Cy5 screen, taken from the pool of hits greater than three standard deviations away from the mean, were purchased or resynthesized and then individually tested for Nsp15 inhibition and fluorescence quenching using both the 5′FAM and 5′Cy5-conjugated substrates. The results of this analysis, shown in Fig. S3, identified eight compounds (CID5220994, CID5266986, CID5675221, CID5326429, hexachlorophene, IPA3, β-lapachone, Reactive Blue 2) that inhibited Nsp15 using either substrate, without appreciable quenching.

### Validation of HTS hits

One of our hits, β-lapachone, had previously been identified in an Nsp15 inhibitor screen (5) and was shown to induce nonspecific enzyme inhibition through production of reactive oxygen species. We confirmed this finding using an Amplex Red assay, which measures hydrogen peroxide formation caused by reducing agents such as DTT undergoing redox cycling in the presence of oxygen or certain compounds (Fig. S4) (39). Consequently, β-lapachone was excluded from further analysis.

Interactions with the substrate or artificial chemical moieties on the substrate can be a source of assay interference (40). To ensure that our compounds could inhibit Nsp15 activity on native substrates, we tested the remaining seven compounds in a PAGE-based RNA cleavage assay. Briefly, this assay employed a 31-nt poly(A) ssRNA substrate containing a single uridylate, which when cleaved by Nsp15 generates RNA fragments of 10-nt and 21-nt in length. We set up cleavage reactions with Nsp15 and native substrates in the absence or the presence of 25 μM or 50 μM of each compound and analyzed the reaction products on a SYBR Gold-stained gel. In our initial assay shown in Fig. S5, three of seven compounds displayed robust inhibitory activity, whereas the others displayed weaker effects. Inhibition of Nsp15 by CID5326429 was modest and sporadic, and Reactive Blue 2 produced a fluorescent artifact, resulting in these compounds being deprioritized. Titrations of the remaining five compounds, CID5220994, CID5266986, CID5675221, hexachlorophene, and IPA-3, using the native substrate assay revealed that all five displayed dose-dependent effects (Fig. 2, *C*–*G*).

PAIN compounds are nuisance molecules that commonly come up as hits in HTSs because of their ability to interfere with common biochemical assays or inactivate enzymes in a promiscuous manner (33). We performed a battery of tests to evaluate if any of our hits displayed these properties. First, we investigated if our lead hits could be forming colloidal aggregates that block Nsp15 activity by carrying out reactions in the presence of a nonionic detergent, to see if inhibition would be relieved (41). As shown in Fig. S6, there was an increase in Nsp15 activity with CID5266986, CID5675221, hexachlorophene, and IPA-3 and a higher increase in activity with CID5220994, when reactions were performed in the presence of either 0.01% Triton-X or CHAPS. However, we also observed a roughly twofold increase in the baseline activity of Nsp15 in the presence of detergents, which accounted for some of the increased enzyme activity observed with the inhibitors. This increase in enzyme activity in response to detergents has previously been documented and is speculated to occur because of reduced adhesion of active enzyme to plastic surfaces in wells (42). Second, we tested if any of our compounds inhibited Nsp15 by inducing protein aggregation and/or denaturation. For this purpose, a dynamic light scattering (DLS) assay that estimates Nsp15 protein particle diameter based on the rate at which scattered light fluctuates in the solution was performed, in the absence or the presence of each of our hits (43). The results of this analysis yielded a single sharp peak on the intensity size distribution plot for all samples, with polydispersity index values ranging from 0.06 to 0.09 and consistent particle diameters ranging from 12.2 to 13.0 nm. These data reflect a monodisperse and homogenous sample (44) and indicate that protein aggregation was not induced by the compounds at the tested concentrations (Fig. S7). Finally, to rule out general RNA–compound interactions, we performed fluorescence polarization assays with an RNA probe in the presence of each of the five compounds. As shown in Fig. S8, we did not detect any appreciable binding between the compounds and the RNA substrate. Collectively, these data imply that CID5220994, CID5266986, CID5675221, hexachlorophene, and IPA-3 inhibit Nsp15 activity *via* nontrivial mechanisms.

### Characterization of lead compounds

**Figure 3:**
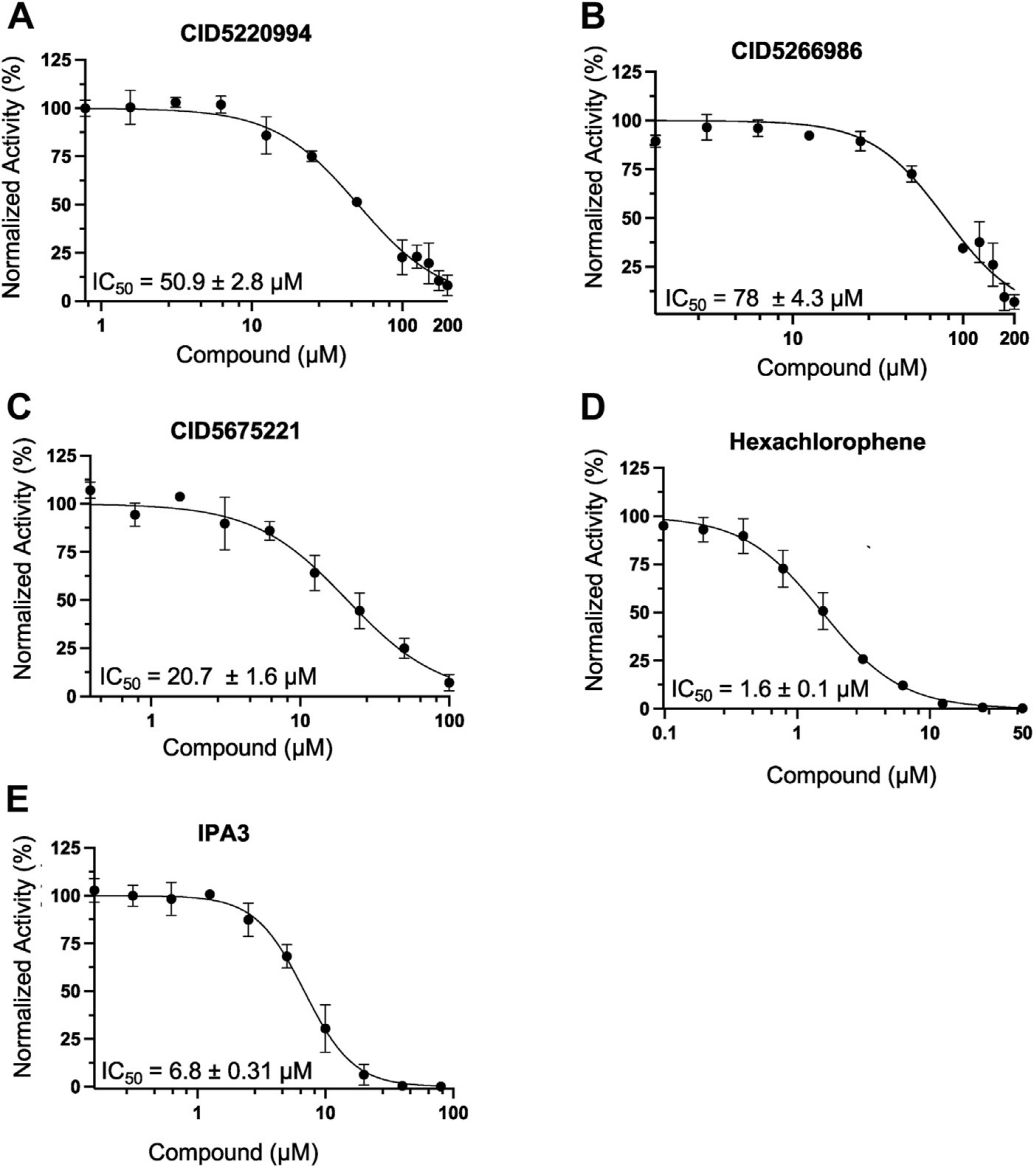
**IC**_**50**_**determination of lead compounds using the FRET-based Nsp15 activity assay.** Plots showing Nsp15 activity in the presence of increasing doses of (*A*) CID5220994, (*B*) CID5266986, (*C*) CID5675221, (*D*) hexachlorophene, or (*E*) IPA-3. Nsp15 (1 ng/μl) and RNA substrate (25 ng/μl) were incubated with compounds for ∼12 min at 37 °C. Values were normalized to activity in the absence of inhibitor. IC_50_ values were calculated using [inhibitor] *versus* normalized response variable slope curves generated by GraphPad Prism (*y* = 100/(1 + (IC_50_/[I])^HillSlope^)); mean ± SD of replicates is shown (n = 3). All experiments were repeated three times with similar results. Nsp, nonstructural protein.

**Figure 4:**
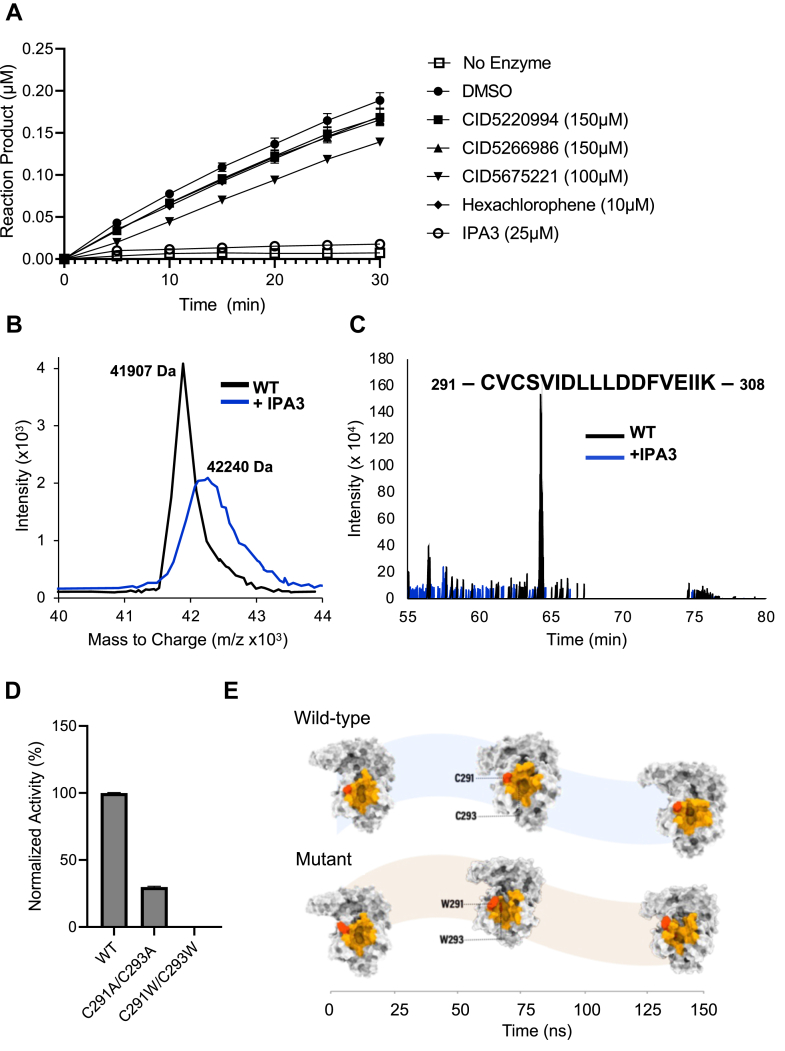
**Assessment of inhibitor reversibility and characterization of covalent modifications imparted by IPA-3.***A*, plot showing Nsp15 activity over time following dilution of inhibitor. Enzyme (1 μg) was preincubated with the indicated concentrations of inhibitors in a cold room for 60 min and subsequently diluted 1:50 into reaction buffer containing 1 μM substrate, at which time fluorescence measurements were begun (*t* = 0). Measurements were taken every 5 min for a total of 30 min. Activity values were transformed to micromolar reaction product using a standard curve. Mean ± SD is shown (n = 3). Points were fit to linear equations. *B*, plot showing intensity *versus* mass to charge ratio distribution for untreated Nsp15 protein (*black*) or Nsp15 protein preincubated with IPA-3 (*blue*), as determined by MALDI-TOF mass spectrometry. The calculated mass is indicated at the top of each central peak. This experiment was repeated three times with similar results. *C*, plot showing intensity *versus* time, determined by LC–MS/MS, for peptides derived from untreated (*black*) or IPA-3-treated (*blue*) Nsp15 protein. The sequence of the peptide corresponding to amino acids 291 to 308 of Nsp15, which was absent in the treated sample, is indicated. *D*, comparison of the activity of wildtype Nsp15 with Nsp15-C291A/C293A and Nsp15-C291W/C293W mutant proteins using the FRET-based assay. Nsp15 (1 ng/μl) was incubated with 1 μM substrate, and reactions were allowed to proceed for 15 min. Activity was normalized to the wildtype protein. Mean ± SD is shown (n = 3). This experiment was repeated three times with similar results. *E*, diagram showing the results of an MD simulation comparing the active site of wildtype Nsp15 protein to that of a C291W/C293W-Nsp15 mutant protein; duration is indicated on the *x*-axis. The active site is colored in *orange*, and residues being substituted are shown in *red*. MD, molecular dynamics; Nsp, nonstructural protein.

To characterize our validated hits, we performed detailed dose–response titrations to quantify the concentration at which each of them inhibited Nsp15 activity by 50% (IC_50_). As shown in Figure 3, CID5220994 and CID5266986 had relatively high IC_50_ values (∼50 and 78 μM), whereas CID5675221 was a stronger inhibitor (IC_50_ of ∼20 μM), and hexachlorophene and IPA-3 were the most potent (IC_50s_ of ∼1 μM and ∼7 μM, respectively). These values were generally consistent with the results of the semiquantitative native substrate assay (Figs. 2 and 3). The fact that CID567221 appears less potent in the gel-based assay than the fluorescent assay can be explained on the basis that it exhibits some quenching in the 5′ FAM channel (Fig. S3). Next, we performed titrations of RNA substrate in the presence of fixed concentrations of each inhibitor to gain insight into their kinetic mechanisms. Modeling these results using steady-state enzyme kinetics (38), we found that CID5266986, CID5675221, and hexachlorophene displayed mixed mechanisms of inhibition (increased *K*_*m*_ and decreased *V*_max_), whereas CID5220994 appeared to be competitive with substrate (increased *K*_*m*_ only), and IPA-3 was noncompetitive (decreased *V*_max_ only, Fig. S9). Calculated *K*_i_ values for the inhibitors ranged from ∼1 to 73 μM (Fig. S9*F*). Finally, to establish if these inhibitors were reversible or irreversible, we obtained reaction progress curves following release of preincubated enzyme–inhibitor complexes by dilution (Fig. 4*A*). These data revealed that Nsp15 activity was restored once concentrations of CID5220994, CID5266986, CID5675221, and hexachlorophene were dramatically lowered, whereas IPA-3 inhibition was irreversible.

Previous studies have characterized IPA-3 as a selective, allosteric, and irreversible inhibitor of the Pak1 kinase (35, 45). IPA-3 contains a central disulfide bond (Fig. 2*G*), and while it does not form mixed disulfides with surface-exposed cysteines on Pak1, it covalently modifies cysteines within its regulatory domain and blocks binding to its upstream activator, Cdc42 (45). To test if the IPA-3 might be inhibiting Nsp15 *via* its reactive disulfide group, we first performed Nsp15 cleavage reactions in the presence of standard (1 mM) and high (10 mM) concentrations of DTT reducing agent. Previous work showed that DTT concentrations greater than 1 mM relieved Pak1 inhibition by IPA-3 (35). In line with these results, we found that excess DTT nearly completely reversed inhibition by IPA-3, implicating thiol groups in its mechanism of inhibition (Fig. S10).

We used TOF mass spectrometry to further characterize the interaction. We found that preincubation of Nsp15 with IPA-3 resulted in a ∼333 Da peak shift *versus* the apoprotein (Figs. 4*B* and S11), corresponding roughly to the molecular weight of one IPA-3 molecule (350 Da). Next, we attempted to map the location of the modified residues using LC–MS/MS. There are a total of five Cys residues within Nsp15, and several of these are known to influence Nsp15 activity (46). While we were unable to detect any modified peptides, we did note the absence of a particular peptide species, corresponding to amino acids 291 to 308 in Nsp15, in the IPA-3-treated sample *versus* the control (Fig. 4*C*). Importantly, this peptide contained two cysteines, Cys291 and Cys293, that were recently shown to form adducts with β-mercaptoethanol (47). Given this, we speculated that Cys291 and Cys293 might be undergoing split IPA-3 modification (half of the structure), which would also explain the corresponding peak shift. Moreover, as these residues are adjacent to Lys290 and Ser294, two key active site residues involved in catalysis (19, 20), we investigated what effects their modification might have on Nsp15 enzyme activity. We generated C291A/C293A and C291W/C293W mutant proteins and found that substitution of these Cys residues with Ala reduced enzyme activity, and that replacement with Trp residues, which sterically mimic the bulky diphenyl of the split IPA-3 modification, inactivated the enzyme (Fig. 4*D*). We analyzed the mutant proteins using size-exclusion chromatography. As shown in Fig. S12, we found that the wildtype protein and both mutants yielded single peaks at similar elution volumes (48). While global changes in structure and hexamerization cannot be fully ruled out as a possible explanation for the loss of activity of the mutants, molecular dynamics (MD) simulations comparing the structure of wildtype Nsp15 (20) to that of a computationally modeled C291W/C293W mutant protein revealed that the bulky residues likely cause a distortion of the active site. This could lead to impaired substrate binding or improper positioning within the catalytic core (Fig. 4*E*). Together, these data indicate that IPA-3 inhibits Nsp15 activity irreversibly through covalent modification of Cys residues, and that Cys291 and Cys293 are likely implicated in the mechanism of inhibition.

**Figure 5:**
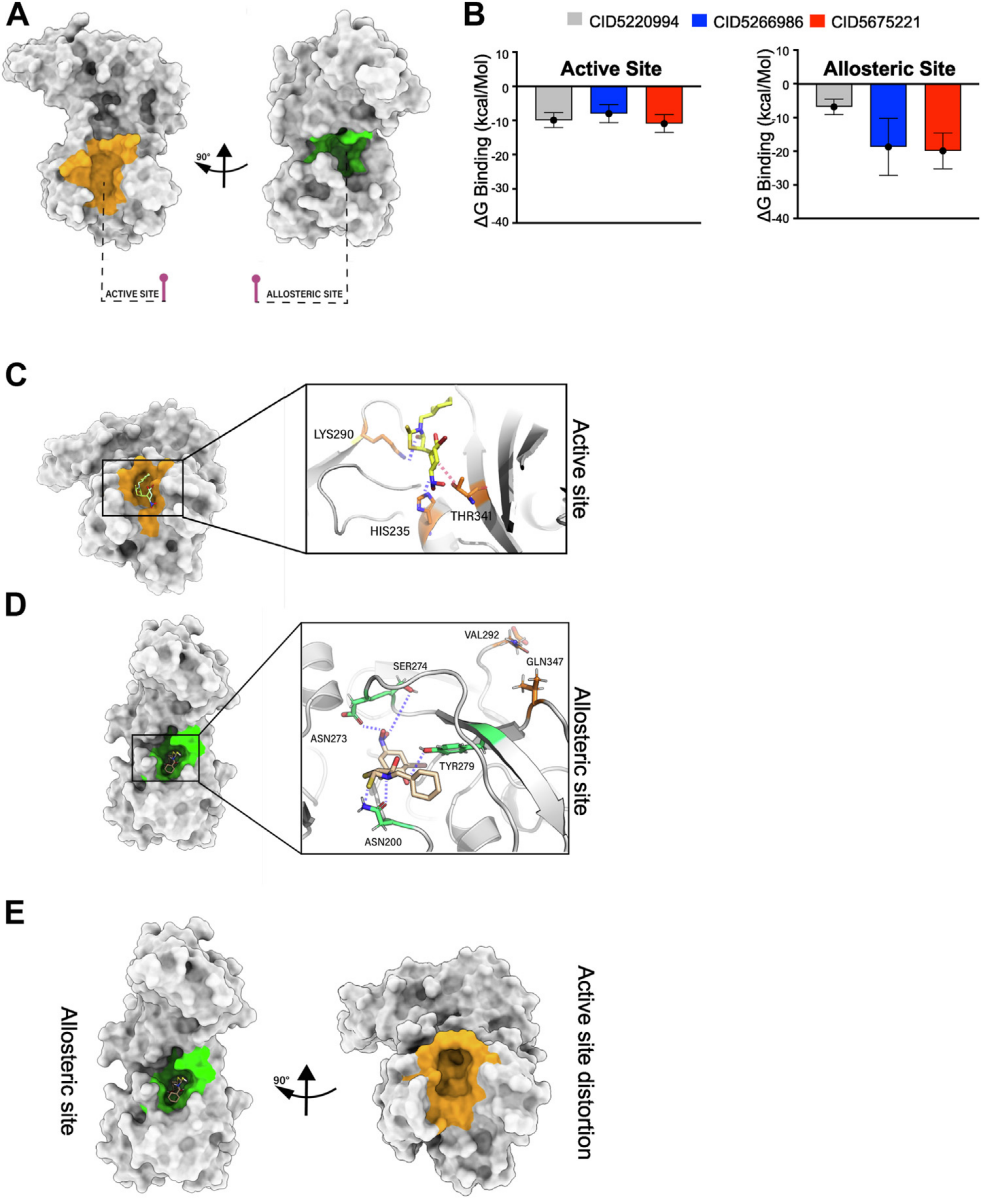
**Binding site analysis and molecular modeling of several lead Nsp15 inhibitors.***A*, protein structures showing the Nsp15 catalytic (active) site colored in *orange* and the identified allosteric site colored in *green*. *B*, free energy of binding for select inhibitors in the active or allosteric pockets. Binding mode of the mixed inhibitor CID5675221 inside the (*C*) catalytic pocket or (*D*) allosteric pocket of Nsp15 after 150 ns of MD simulation. Hydrogen bonds and hydrophobic contacts are shown using *dashed blue* and *red lines*, respectively. *E*, diagram highlighting the active site distortion caused by binding of CID5675221 into the allosteric site. MD, molecular dynamics; Nsp, nonstructural protein.

We attempted to cocrystallize the reversible inhibitors with Nsp15 to gain deeper insight into their mechanisms. While we were able to generate a novel structure of an Nsp15-H250A catalytic mutant protein (26) (Figs. 1*B* and S13), we were unable to obtain structures for the Nsp15–inhibitor complexes. Therefore, we used molecular modeling and MD simulation studies as an alternative strategy. In addition to the previously reported active site (20), we identified a deeper allosteric binding site on the surface of the enzyme that could mediate interactions with the noncompetitive inhibitors (Fig. 5*A*). Several residues within this pocket including Tyr279 have previously been implicated in chemical interactions with Exebryl-1 (5) and other Nsp15 inhibitors (32). Initially, we docked the four reversible inhibitors into both the active and allosteric sites in order to determine their differential affinity for each site. Subsequently, Nsp15–inhibitor complexes were subjected to a 150 ns MD simulation in order to investigate the binding dynamics and free energies of each inhibitor in both active and allosteric sites. In agreement with our experimental data, the MMPBSA binding affinity of the competitive inhibitor CID5220994 was higher in the active site compared with the allosteric site (Fig. 5*B*). Interestingly, the mixed inhibitors (CID5266986 and CID5675221) exhibited affinity for both binding sites, although the affinity for the allosteric site was significantly higher (Fig. 5*B*). Unfortunately, we were unable to model hexachlorophene binding because of its chlorine atoms interfering with the total simulation system charges. Binding modes of CID5675221 into the active site and allosteric site are shown in Figure 5, *C* and *D*, with the resulting active site distortion highlighted in Figure 5*E*. Depictions of the binding modes of CID5220994 and CID5266986 are presented in Fig. S14. Collectively, these data provide a theoretical basis for the kinetic mechanisms of the lead compounds and provide structural insight into how these molecules may be inhibiting Nsp15 activity.

### Assay of ability of lead compounds to suppress SARS-CoV-2 replication in cells

**Figure 6:**
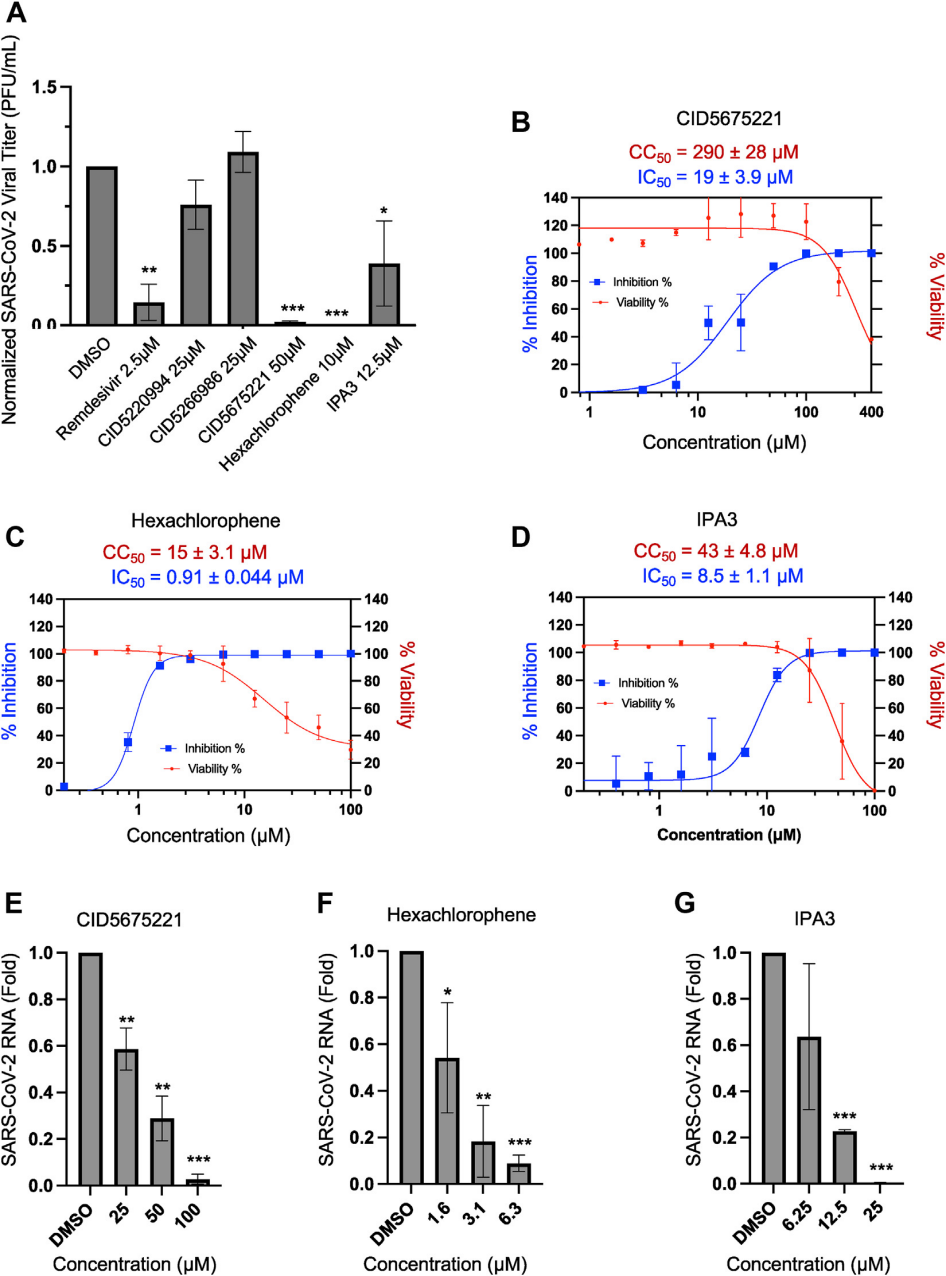
**Determination of anti-SARS-CoV-2 activity of Nsp15 inhibitors in cells.***A*, bar graph showing SARS-CoV-2 replication, as determined by viral plaque assays, in the absence or the presence of Nsp15 inhibitors. Vero CCL-81 cells were infected with SARS-CoV-2 (MOI = 0.1) and treated with a single concentration of remdesivir (positive control), CID5220994, CID5266986, CID5675221, hexachlorophene, IPA-3, or vehicle (DMSO). Titers were determined by plaque assay of supernatants collected 24 h postinfection and normalized to the DMSO control. Mean ± SD is shown (n = 2). Plots showing the effect of increasing concentrations of (*B*) CID5675221, (*C*) hexachlorophene, or (*D*) IPA-3 on viral inhibition (*blue curve*), and cell viability (*red curve*). The % inhibition and % viability were calculated relative to a DMSO control sample. Viral titers were determined by plaque assay following a 24 h infection of Vero CCL-81 cells infected with SARS-CoV-2 (MOI = 0.1) in the absence or the presence of compounds. Cell viability was assessed at the same time point by measuring the intracellular ATP levels. IC_50_ and CC_50_ values are indicated above the curves and were calculated by fitting points to a four-parameter logistic curve (*y* = bottom + ([I]^HillSlope^) ∗ (top − bottom)/([I]^HillSlope^ + IC_50_^HillSlope^)) using GraphPad Prism. Mean ± SD of two biological replicates is shown. Effect of the indicated doses of (*E*) CID5675221, (*F*) hexachlorophene, or (*G*) IPA-3 on levels of SARS-CoV-2 viral RNA in Vero CCL-81 cells as determined by qRT–PCR from total cellular RNA following a 24 h infection with SARS-CoV-2 (MOI = 0.1). Mean ± SD (n = 2) is shown. ∗ denotes *p* < 0.05, ∗∗ denotes *p* < 0.01, and ∗∗∗ denotes *p* < 0.001 significance using a one-tailed Student's *t* test *versus* the DMSO control sample. DMSO, dimethyl sulfoxide; MOI, multiplicity of infection; Nsp, nonstructural protein; qRT–PCR, quantitative RT–PCR; SARS-CoV-2, severe acute respiratory syndrome coronavirus 2.

To translate our findings to cells, we first evaluated the toxicity of the five lead compounds using a luminescent ATP-based cell viability assay (CellTiter-Glo) in Vero CCL-81 cells. These are monkey kidney cells that have previously been used as a model for SARS-CoV-2 infection (49). CID5675221 had a very high CC_50_ value (>240 μM), whereas CID5220994, CID5266986, hexachlorophene, and IPA-3 were moderately toxic to cells at higher doses, with CC_50_ values ranging from ∼15 to 42 μM (Fig. S15). Next, we used a plaque assay to test the ability of these compounds to inhibit production of infectious SARS-CoV-2 virions at subtoxic concentrations; remdesivir was used as a positive control. As shown in Figure 6*A*, CID5675221, hexachlorophene, IPA-3, and the positive control significantly reduced viral titers, whereas the effects of CID5220994 and CID5266986 were not statistically different from the control.

To establish the approximate selectivity indexes for the three bioactive compounds, we performed dose titrations and measured their inhibitory effects on viral replication and cell viability in parallel. Cellular IC_50_ values were ∼20 μM, ∼1 μM, and 10 μM for CID5675221, hexachlorophene, and IPA-3, respectively (Fig. 6, *B*–*D*), mirroring our *in vitro* results (Fig. 3, *C*–*E*). Using these data in combination with the cytotoxicity measurements (Fig. 6, *B*–*D*), we determined the selectivity index of the small molecules to be CID5675221 ∼15, hexachlorophene ∼16, and IPA-3 ∼5. Finally, as complementary assays for assessing virus replication and infection, respectively, we measured intracellular SARS-CoV-2 RNA using quantitative RT–PCR and assessed levels of the viral spike protein in cells using immunofluorescence in the absence or the presence of each compound. We found that all three compounds significantly reduced viral RNA in a dose-dependent manner (Fig. 6, *E*–*G*) and decreased the presence of viral spike proteins in Vero CCL-81 cells (Fig. S16). These findings establish several new Nsp15 inhibitors that actively inhibit SARS-CoV-2 replication in cells at nontoxic doses.

### Selectivity of inhibitors against Nsp15 homologs and unrelated proteins

Nsp15 from SARS-CoV-2 is highly similar to homologs from SARS-CoV-1 and Middle East respiratory syndrome (MERS) (Fig. S17), displaying amino acid identity of 88% and 51%, respectively (20). Based on this, we wondered if our lead inhibitors might also be effective in inhibiting these related enzymes. We tested the ability of the five lead compounds to inhibit the Nsp15 homologs from these viruses at two doses corresponding roughly to the SARS-CoV-2 IC_50_ and IC_100_ values. In agreement with our hypothesis, we found that the effects of the compounds on Nsp15 from SARS-CoV-1 were virtually equivalent to those observed with the SARS-CoV-2 enzyme, with the exception of hexachlorophene, which was less potent (Fig. S18*A*). We also observed some degree of inhibition of MERS Nsp15 by all lead compounds, albeit with reduced potency. This was especially evident for CID5220994 and CID5266986, and to some extent IPA-3, which displayed only modest effects (Fig. S18*B*). We next tested if these compounds could inhibit the activity of RNAse A, a more distantly related RNA endonuclease that uses a similar catalytic mechanism. As shown in Fig. S18*C*, we did not observe any inhibition of this enzyme by the compounds. Finally, we tested the effects of the lead compounds on a completely unrelated enzyme, the NAD-dependent lysine deacetylase SIRT1 (40). As anticipated, none of the compounds inhibited SIRT1 enzymatic activity (Fig. S18*D*). In sum, these data show that CID5220994, CID5266986, CID5675221, hexachlorophene, and IPA-3 are able to selectively inhibit Nsp15 homologs from multiple coronaviruses without altering the activity of other RNA endonucleases and unrelated enzymes.

## Discussion

Outbreaks of SARS-CoV-2 around the world continue, and there remains a need to develop new therapeutics to ensure that treatments against evolved variants are available (1). From a screen of over 100,000 small molecules, we identified five promising lead compounds that inhibit Nsp15 activity in a native substrate assay *in vitro* (Fig. 2). Three of these, hexachlorophene, IPA-3, and CID5675221, were effective in blocking SARS-CoV-2 viral replication in cells (Fig. 6). The results of this study add to the growing body of evidence (5, 32) that suggests fully optimized Nsp15 inhibitors could 1 day be employed as alternative or complementary therapeutics to current state-of-the-art COVID-19 drugs.

Lipinski’s rule of 5 is a classic predictor of how suitable a molecule may be as a drug (50). This theory states that a drug-like molecule should (1) have no more than five hydrogen bonds, (2) have no more than ten hydrogen bond acceptors, (3) have a molecular weight of <500 Da, and (4) have a lipophilicity (LogP) value of <5 (50). As shown in Fig. S19, our five lead compounds fulfilled all these criteria, with the exception of hexachlorophene, which had a larger LogP value of 7.25. However, our empirical data identified several properties of these first-generation inhibitors that would need to be revamped to realize their therapeutic potential. For example, both CID5220994 and CID5266986, the two weakest inhibitors, caused toxicity in cells at concentrations close to their *in vitro* IC_50_ values (Figs. 3, 6 and S15). Interestingly, CID5266986 bears striking structural similarity to Exebryl-1, a compound currently in clinical trials for the treatment of Alzheimer’s disease that was recently characterized as a mixed inhibitor of Nsp15 (5).

Hexachlorophene (Nabac) has a long history of use as a biocide in toothpaste, soaps, and topical treatments, although it has recently been removed from many nonprescription products because of concerns over neurotoxicity (51). Its antimicrobial activity results from its ability to inhibit the membrane-bound part of the electron transport chain, leading to inhibition of respiration and cell leakage (52). In addition, it inhibits a broad range of different enzymes, including phosphatases such as SHP2 (53), adenylyl cyclases (54), and succinate dehydrogenase (55). Our data show that hexachlorophene also potently inhibits the Nsp15 enzyme and blocks SARS-CoV-2 viral replication in cells with an IC_50_ of ∼1 to 2 μM (Figs. 3 and 6). While we are the first to report inhibition of Nsp15 by this compound, several past studies have implicated hexachlorophene in related antiviral activity. For example, a previous publication showed that hexachlorophene abrogated SARS-CoV-2 replication in Vero cells (56). Moreover, a separate publication characterized hexachlorophene as a competitive inhibitor of the 3CL protease from SARS-CoV-1 (57). While its promiscuity, toxicity, and chemical properties preclude it from being used systemically, our findings suggest that hexachlorophene could be a promising additive to topical formulations aimed at reducing the spread of COVID-19 *via* hand and skin sanitization. For example, the prescription skin cleaner pHisoHex contains a 3% w/w emulsion of hexachlorophene, which corresponds to a concentration of 73.7 mM (58). While topical agents using this dose carry some risk because of absorption and toxicity (51), our results indicate that a dose ∼10,000 times lower (Fig. 6) would be sufficient to block SARS-CoV-2 replication.

SARS-CoV-2 Nsp15 contains five cysteine residues (Cys103, Cys117, Cys291, Cys293, and Cys334) that have been implicated in subunit oligomerization (59) and interactions with the RNA substrate (47). Our finding that IPA-3 inhibits Nsp15 activity through potential covalent modification of cysteines is supported by previous work demonstrating the same mode of action against the Pak1 kinase (60). While there is a ∼16 to 18 Da discrepancy between the sum of the masses of Nsp15 and IPA-3 (42,257 Da) and the experimentally determined mass for the main Nsp15-IPA-3 adduct (42,240 Da), this could be explained by the loss of one hydroxyl group on IPA-3, alone, or in combination with the loss of reduced cysteine hydrogens on the protein during covalent reaction. However, further investigation is needed to elucidate the exact chemistry of the reaction. Our assertion that Cys291 and Cys293 (Fig. 4) play a crucial role in this process is supported by a previous cryo-EM study that designated these residues as reactive (47). Moreover, modeling from this study indicated that Cys291 may participate in substrate interactions in the postcleavage state of the enzyme (47). Our independent docking and simulation studies show that modification of these residues by bulky aromatic groups, such as those found within IPA-3, causes a distortion in the active site that may impair substrate positioning and/or release (Fig. 4*E*). This finding was confirmed experimentally by our mutation studies showing that substitution of these residues with tryptophan results in a loss of enzyme activity (Fig. 4*D*). Finally, the fact that IPA-3 inhibits Nsp15 from SARS-CoV-1 and SARS-CoV-2 with equal potency, but shows reduced effect on the MERS homolog (Fig. S18), could be explained on the basis of the conservation of both Cys291/Cys293 amongst the SARS viruses but only Cys293 in MERS (Fig. S17).

IPA-3 has previously been characterized as an isoform-selective non-ATP competitive inhibitor of the p21-activated kinase Pak1, and it is currently being investigated as a potential anticancer drug (35, 60). While initial reports showed that IPA-3 did not react with surface-exposed cysteines on Pak1 (35), later reports demonstrated its mechanism of inhibition to be covalent modification of the protein regulatory domain (45). Covalent inhibitors bind to targets in two distinct steps: (1) equilibrium bond formation or reversible interaction and (2) covalent bond formation or irreversible interaction (61). Despite previously being classified as PAIN molecules (62), modern covalent inhibitors are emerging with advantages over traditional mechanistic inhibitors, including increased potency, the ability to target shallow binding sites, and defense against drug resistance (61). In fact, the recently approved COVID-19 drug paxlovid acts through reversible covalent inhibition of the 3CL protease (63). Although IPA-3 inhibits multiple enzymes and is not specific for Nsp15 (35), medicinal chemistry work could be performed to refine its binding selectivity. Moreover, the novel mechanism of covalent inhibition of Nsp15 involving Cys291/293 discovered in this study could be exploited to develop new rationally designed Nsp15 inhibitors.

CID5675221 is a novel compound based on a rhodanine scaffold, which is found in drugs such as the aldose reductase inhibitor epalrestat (64). This small molecule demonstrated efficacy against SARS-CoV-2 with an IC_50_ of <20 μM and was the least toxic of any lead hit in our study (Figs. 6 and S15). Our kinetic experiments revealed that this compound had an apparent mixed mode of inhibition (Fig. S9). However, our structural studies suggest a primarily noncompetitive mechanism of inhibition for CID5675221, stemming from the formation of multiple hydrogen bonds with residues located in the N-terminal allosteric pocket, including Tyr279, Asn200, Ser274, and Asn273 (Fig. 5, *D* and *E*). We observed that binding of CID5675221 to the allosteric site distorts Gln347, an essential residue of the Nsp15 catalytic site (Fig. 5, *D* and *E*). More precisely, CID5675221 exerts structural strain on Gln347, shortening the distance between it and Val292 from 16.6 to 4.8 Å, causing closure of one side of the catalytic site (Fig. 5*E*). While we speculate that CID5675221-mediated Nsp15 inhibition is caused mostly by binding to the allosteric and not the active site, our observations during the MD simulation do account for mixed inhibitory effects (Fig. 5).

Based on this model, several refinements to the chemical structure of CID5675221 can be proposed. Foremost, we propose eliminating the nitro group from the molecule, as this moiety has classically been considered a toxicophore by medicinal chemists (65). Our *in silico* model indicates that bioisosteric replacement of this group with a carboxylic acid would not alter molecular stability or affinity of the inhibitor for the allosteric pocket. In addition, we hypothesize that introduction of a hydroxyl group to the ortho position of the benzene ring, resulting in a hydrogen bond gain, would impose additional strain on Tyr279 and Gln347. While these modifications are only based on preliminary structural studies, we speculate that CID5675221 is an attractive lead hit for further development using medicinal chemistry approaches.

Overall, this study identifies a set of five lead inhibitors for SARS-CoV-2 Nsp15, a promising coronavirus drug target (29), that operate through diverse reversible and irreversible (covalent) mechanisms. We propose that derivatization of these molecules through chemistry, supported by additional crystallographic studies, and/or rational design of new molecules that harness the mechanisms described in this work, could lead to second-generation inhibitors with improved potency and drug-like properties. As many of these scaffolds are active against Nsp15 variants from other coronaviruses (Fig. S18), optimized Nsp15 inhibitor therapeutics would be useful not only for the treatment of COVID-19 but also infections from SARS-CoV-1, MERS, and related coronaviruses that are known or yet to be discovered.

## Experimental procedures

### Constructs and cloning

The sequence encoding wildtype SARS-CoV-2 Nsp15 protein (Protein Data Bank [PDB] ID: 6VWW) (20) was codon optimized for bacterial expression and purchased as a custom gene synthesis plasmid from IDT (Table S1). This sequence was PCR amplified using primers found in Table S2 and inserted into a pET-based vector (derived from pC013; Addgene #90097) using the NEBuilder HiFi DNA assembly cloning kit (NEB), according to the manufacturer’s instructions. The resulting construct was verified by Sanger sequencing using T7 primers (Table S2). The Δ0–28 Δ336–347 Nsp15 mutant construct was prepared in a similar fashion using its specific primers (Table S2). To prepare the Nsp15 H250A, C291A/C293A, and C291W/C293W mutants, Q5 site-directed mutagenesis (NEB) was performed on the wildtype Nsp15 construct using the primer sequences included in Table S2, as per the manufacturer’s instructions. Sequences corresponding to SARS-CoV-1 Nsp15 and MERS Nsp15 were ordered as bacterial codon-optimized gBlocks from IDT (sequences in Table S1) and were cloned into the BamHI and HindIII restriction sites of a pET-28B(+) plasmid. All plasmids were validated by sequencing.

### Nsp15 protein purification

Wildtype and mutant Nsp15 proteins were purified as previously described (20), with several modifications. Briefly, BL21(DE3)pLYsS cells were transformed with plasmids encoding Nsp15. Starter cultures were grown overnight (∼16 h) at 37 °C with 5 ml of LB broth in the presence of 50 μg/ml carbenicillin. About 1 ml of this culture was then used to inoculate 2 l of LB–carbenicillin, and this culture was grown as above until an absorbance of 0.6 at 600 nm was reached. Following this, IPTG was added to a final concentration of 1 mM, and the culture was incubated at 18 °C overnight. Cells were pelleted by centrifugation at 3500*g* for 15 min and resuspended in lysis buffer (20 mM Tris–Cl, pH 7.5, 250 mM NaCl, 5 mM imidazole, pH 8.0) supplemented with Roche Complete Ultra protease inhibitor (Sigma) and 0.1 M PMSF. The mixture was incubated on ice for 30 min before being sonicated (15 s pulse-on and 59 s pulse-off for a total of 15 min at 55% amplitude). Cellular debris was pelleted by centrifuging at 28,000*g* for 1 h. Next, the lysate was collected and subjected to filtering through a 0.45 μM polyvinylidene difluoride membrane. The filtered lysate was loaded onto a 1 ml HisTrap HP column (Sigma) and purified using an AKTA Start System (Cytiva). The column was washed with buffer (20 mM Tris–Cl, pH 8.0, 250 mM NaCl, 10 mM imidazole, pH 8.0) until UV baseline was reached and subsequently eluted in gradient fashion. The final elution buffer was comprised of 20 mM Tris–Cl, pH 8.0, 250 mM NaCl, 250 mM imidazole, pH 8.0. Pooled protein fractions were concentrated with a Pierce Protein concentrator 10K (Thermo). During concentration, the buffer was exchanged with 20 mM 30 Hepes–KOH, pH 7.5, 500 mM NaCl, 1 mM DTT, and 10% glycerol. Concentrated protein was aliquoted and stored at −80 °C until usage. Protein concentration was measured using the DTT-resistant Pierce 660 nM Protein BCA Assay kit (Thermo).

### Nsp15 FRET-based activity assay

We adapted a previously described FRET-based assay, which employs a uracil-containing RNA substrate that is flanked by fluorophore and quencher moieties (21). Sequences for the substrates are listed in Table S3. Reactions were set up in black 96-well flat-bottom polystyrene plates (Corning) in 60 μl volume of reaction buffer (25 mM Hepes, 50 mM NaCl, 5 mM MnCl_2_, and 1 mM DTT) and contained 1 ng/μl Nsp15 and 1 μM substrate unless otherwise stated. Where applicable, compounds were dissolved in DMSO and subsequently added to the reaction (DMSO concentration was kept to less than 1%). Reactions were incubated at 37 °C for the indicated times and read at excitation/emission wavelengths of 490/520 nm for FAM or 645/670 nm for Cy5 using a SpectraMax i3x spectrophotometer (Molecular Devices).

### HTS

Screening was performed at the High Content Analysis Core facility at the University of Alberta and at the Biofactorial High Throughput Biology Facility at the University of British Columbia. The library of compounds screened was comprised of roughly 30,000 compounds from the Canadian Chemical Biology Network collection, 1280 from LOPAC, 3040 from the TimTec collection, 50,000 from the ChemBridge DIVERSet collection, and 24,000 ChemBridge compounds from the GlycoNet collection. Compound overlap between the collections was <0.1%. Reagents were distributed into 384-well black flat bottom plates (Greiner) using either a JANUS 384-well liquid handling system (PerkinElmer) or an Echo525 acoustic dispenser (Beckman Coulter) contained in a Labcyte Access Workstation. The reaction setup was similar to that described previously, in which the final concentration of Nsp15 was 1 ng/μl and that of RNA was 0.5 μM. The reaction volume was 20 μl. Sequences for the positive control, RNA2, and Cy5 RNA substrates are listed in Table S3. Compounds were dissolved in DMSO and screened at a final concentration of roughly 10 μM. DMSO without any inhibitors was used as a negative control, and either 100 μM of benzopurpurin B or a reaction without Nsp15 was used as a positive control. Readings were taken at excitation/emission wavelengths of 490/520 nm for FAM or 645/670 nm for Cy5. Read 1 measured autofluorescence in a reaction mixture containing buffer, Nsp15, and compound. Subsequently, RNA substrate was added, and following incubation at 37 °C in a humidified incubator for 20 min, the reaction was stopped by addition of 100 mM EDTA and read 2 was performed. After the addition of 1 μM of positive control FAM-RNA (Table S3) to the reaction, read 3 was taken to test for potential quenching. Percent inhibition values were calculated as follows: (read 2 − read 1)/average negative control. Compounds that were found to quench the positive control by >50% in read 3 were excluded from further analysis.

### Amplex Red assay

Redox reactivity of the lead compounds was assessed using the Amplex Red Hydrogen Peroxide/Peroxidase Assay Kit (Invitrogen), according to the manufacturer’s instructions. Briefly, test compounds were diluted in assay buffer to a final concentration of 100 μM in the presence of the indicated concentrations of DTT in 96-well plates. Hydrogen peroxide at a concentration of 10 μM was used as a positive control. The reaction was started by addition of 0.2 U/ml horseradish peroxidase and 50 μM final concentration of Amplex Red reagent. The reaction was incubated in the dark for 15 min at room temperature. Plates were read using a SpectraMax i3x spectrophotometer at excitation and emission wavelengths of 560 nm and 590 nm, respectively.

### Nsp15 native RNA cleavage assay

The native RNA cleavage assays employed a 31 nucleotide (nt) ssRNA (IDT and Biosynthesis) with the sequence 5′-rArArArArArArArArArArArArArArArArArArArArUrArArArArArArArArArA-3′, whose cleavage at the “rU” site results in 21 nt and 10 nt fragments. The reaction was set up in a 10 μl volume containing a final concentration of 7.5 ng/μl Nsp15 and 250 ng of RNA in assay buffer (25 mM Hepes, 50 mM NaCl, 5 mM MnCl_2_, and 1 mM DTT) and allowed to run for the indicated time. Prior to gel loading, samples were prepared with 2× formamide-based RNA loading dye (NEB) and were boiled at 95 °C for 5 min. Samples were electrophoresed on a denaturing 15% Mini-Protean TBE–UREA polyacrylamide gel (Bio-Rad) at 200 V for ∼40 min at room temperature. Gels were stained with SYBR Gold (Thermo Fisher) for 20 min and imaged at Cy3 fluorescent channels (520/605 nm excitation/emission) with an Amersham Imager 680 (Cytiva).

### DLS

DLS was performed with a solution containing 1 mg/ml of wildtype Nsp15 in storage buffer (50 mM Hepes [pH 8], 150 mM NaCl, and 1 mM Tris(2-carboxyethyl)phosphine along with a 1% DMSO), in the absence or the presence of the indicated compounds. Compounds were tested at a concentration of fivefold excess compared with protein, with a consistent 1% DMSO concentration. The solutions were spun down and subjected to analysis using a Zetasizer Ultra Red (Malvern Panalytical) instrument in reusable 50 μl cuvettes. Measurements were performed using a He–Ne laser emitting at 633 nm, at a scattering angle of 90°, at a temperature of 25 °C, and with a laser power of 10 mW. Samples were analyzed in three cycles, and the correlation function graphs were averaged. The particle’s hydrodynamic diameter was derived from the correlation function using ZS Xplorer software (Malvern Panalytical, version 1.0).

### Fluorescence polarization assay

Increasing concentrations of Nsp15 protein or inhibitor were titrated against a constant concentration of RNA substrate. A labeled RNA substrate (20 nM) was used in a reaction volume of 20 μl and dispensed in OptiPlate 384 F black microplates (PerkinElmer) after a 10 min incubation. 6-FAM fluorescence was excited at 480 nm, and its emission was measured at 535 nm using an EnVision 2103 Multilabel Plate Reader (PerkinElmer, Inc). The change in polarization was plotted against the logarithmic concentration of the protein or inhibitor.

### MALDI-TOF mass spectroscopy

About 20 mg/ml of wildtype Nsp15 protein was preincubated overnight in the absence or the presence of a fivefold molar excess of each compound. Samples were diluted 10-fold (50% acetonitrile/water + 0.1% trifluoroacetic acid), and 1 μl of each was then mixed with 1 μl of sinapinic acid (10 mg/ml in 50% acetonitrile/water + 0.1% trifluoroacetic acid). The sample–matrix solutions were spotted onto a stainless-steel target plate and allowed to air dry. Mass spectra were acquired using an Autoflex Speed MALDI-TOF mass spectrometer (Bruker Daltonik) with a Smartbeam-II laser at a frequency of 2 KHz. Ions were analyzed in positive mode, and external calibration was performed using a standard protein mixture.

### LC–MS/MS mapping of modified peptides

Samples of recombinant Nsp15 protein (10 μg) were incubated in a buffer solution (25 mM Hepes/pH 7.5 with 50 mM NaCl, 5 mM MnCl_2_, 0.1 mM DTT) in the absence or the presence of 67 μM IPA-3 for periods of either 4 h or 16 h, at 25 °C or 4 °C, respectively. Subsequently, samples were prepared for LC–MS/MS analysis. Samples were dissolved in 100 mM ammonium bicarbonate, reduced (DTT), alkylated (iodoacetamide), and subjected to digestion with trypsin (Promega sequencing grade) overnight at 37 °C. The pH of the samples was then adjusted to 3 to 4 with formic acid, and they were dried, dissolved in water + 0.2% formic acid, and desalted (Pierce C18 tips). The tryptic peptides were resolved and ionized by using nano flow HPLC (Easy-nLC 1000; Thermo Scientific) coupled to a Q Exactive orbitrap mass spectrometer (Thermo Scientific) with an EASY-Spray capillary HPLC column (catalog no.: ES902; Thermo Scientific). The mass spectrometer was operated in data-dependent acquisition mode, recording high-accuracy and high-resolution survey orbitrap spectra using external mass calibration, with a resolution of 35,000 and an *m/z* range of 300 to 1700. The 12 most intense multiply charged ions were sequentially fragmented by using high-emergy collisional dissociation, and spectra of their fragments were recorded in the orbitrap at a resolution of 17,500.

### X-ray crystallography

Crystals of the Nsp15 H250A mutant protein were grown by sitting drop vapor diffusion with a reservoir solution containing 0.2 M calcium acetate, 0.1 M imidazole, HCl (pH 8), and 10% (w/v) PEG 8000. Diffraction data were collected on a home source rotating anode, and indexed, integrated, and scaled using HKL2000 (HKL Research Inc). Molecular replacement was carried out using PHASER (search model PDB ID: 6VWW (https://www.rcsb.org/structure/6VWW), chain A). Data quality assessment was carried out using Xtriage (Phenix). Twinning was detected (Twin law: h, -h-k, and -l), and the twinning law was applied in further refinement steps. Refinement in PHENIX utilized automated noncrystallographic symmetry and reference model restraints throughout, with the latter being removed for the final refinement round.

### Molecular modeling

The crystal structure of Nsp15 was obtained from the PDB (PDB ID: 6WXC) (30), and Schrodinger’s Mastro and EPIK (66) were used to prepare the protein structure by removing chain B, followed by a short minimization, addition of missing side chains, and equilibration of the protonated group to the biological pH (7.0). The SiteMap module of Schrodinger (67) was used to detect binding sites on the surface of Nsp15 protein. The 3D structures of the inhibitors were prepared for docking using the UCSF CHIMERA, V1.10.2 dock prep tool in the framework of the AMBER99SB force field (68). Using AutoDock Vina (69), all the compounds were docked into the two identified binding pockets with a grid box of 40 × 40 × 40 and a spacing of 0.375 Å. A total of 12 docking runs were performed with exhaustiveness of 40 for each inhibitor. Prior to doing the molecular dynamic simulation on the enzyme–inhibitor complexes, an MD simulation on the apoprotein was performed using the GROMACS 2021 package (70). TIP3P water models were utilized to solvate the protein with a 1 nm marginal cushion on each side. The box was then neutralized using NaCl, and the system was minimized using the AMBER99SB0ILDN force field. The system was heated to 300 K and equilibrated for 500 ps using the Berendsen Thermostat. Using the isothermal–isobaric ensemble at 1 bar with the Parrinello–Rahman barostat, an additional equilibration was also performed. A 150 ns production run was performed using the periodic boundary condition. The Lenard–Jones, the Coulomb (cutoff = 1.0 nm), and the particle mesh Ewald were used to calculate the Van Der Waals and electrostatic interactions. The enzyme–inhibitor complexes were analyzed using the same conditions. The AnteChamber Python Parser interfacE (71) was used for ligand parameterization. All the data were plotted using Schrodinger’s PyMOL package and ChimeraX (72). The free energy of interaction between each inhibitor and Nsp15 was calculated using the gmx_MMPBSA tool (73). The C291W/C293W mutant protein structure was generated and prepared using Schrodinger’s Maestro. MD simulation for this protein was performed as described previously, and snapshots were taken every 75 ns for structural comparison, assessment, and analysis.

### Vero CCL-81 cell culture

Cells were obtained from American Type Culture Collection and were previously authenticated and shown to be negative for mycoplasma at the time of purchase. Cells were maintained in high glucose Dulbecco’s modified Eagle's medium (Thermo Fisher) supplemented with 10% fetal bovine serum, Canadian origin (Sigma), and 1× penicillin–streptomycin–glutamine (Thermo), and grown in a 37 °C humidified incubator with 5% CO_2_.

### Cell cytotoxicity assay

Vero CCL-81 cells were seeded in 96-well plates (Greiner) at 10,000 cells per well overnight before compounds were added to wells at the indicated concentrations alongside a DMSO control. Twenty-four hours later, cell viability was assayed using the CellTiter-Glo Luminescent Cell Viability Assay (Promega), according to the manufacturer’s instructions. Briefly, this assay relies on the quantification of ATP using a proprietary Ultra-Glo Luciferase that converts luciferin to luminescent oxyluciferin in the presence of ATP. Cells were incubated in 100 μl of complete media with 100 μl of reconstituted CellTiter-Glo Reagent (buffer plus substrate). Samples were mixed by shaking for 10 min, and then luminescence was measured using a Spectramax i3x (Molecular Devices) device. Data from experimental wells were normalized to the appropriate DMSO control.

### SARS-CoV-2 sourcing and propagation

The Canadian clinical isolate of SARS-CoV-2 72B/CA/CALG (74) was propagated at the biosafety level 3 laboratory at the University of Alberta in Vero-E6 cells (American Type Culture Collection), grown in Dulbecco’s modified Eagle's medium supplemented with 3% fetal bovine serum, 15 mM Hepes, 1× l-glutamine, and penicillin–streptomycin. All experiments using live virus were carried out at this facility, in accordance with approved protocols.

### SARS-CoV-2 plaque assay

SARS-CoV-2 supernatant samples were serially diluted (10-fold dilutions) in fresh media and used to infect monolayers of 1 × 10^5^ Vero-CCL-81 cells in 24-well plates (Greiner) for 1 h. Subsequently, viral supernatants were removed, and cell monolayers were overlaid with a mixture of MEM (Thermo Fisher Scientific) and 0.75 to 1.5% carboxymethylcellulose (Sigma–Aldrich). Cells were maintained at 37 °C for 3 days to allow plaque development to occur. Before plaque counting, cells were fixed with 10% formaldehyde and stained with 1% crystal violet in 20% ethanol.

### Assessment of SARS-CoV-2 RNA levels following drug treatment

Vero CCL-81 cells were seeded in 96-well plates (Greiner) at 1 × 10^4^ cells per well. The next day, cells were rinsed once with PBS, and SARS-CoV-2 at a multiplicity of infection of 0.1 was added to the wells using fresh media supplemented with 10% fetal bovine serum containing either DMSO or inhibitors. The cells were then incubated at 37 °C for 24 h. Next, following removal of the supernatant, total RNA from Vero CCL-81 cells was extracted using the NucleoSpin RNA kit (Macherey–Nagel), following the manufacturer’s protocol. Total RNA was reverse transcribed using 0.5 to 1 μg of total RNA and ImProm-II Reverse Transcriptase (Promega), according to the manufacturer’s protocol. Quantitative RT–PCR was performed with PerfeCTa SYBR Green SuperMix (Quanta BioSciences) using a CFX96 Touch Real-Time PCR Detection System (Bio-Rad). The cycling conditions were 45 cycles of 94 °C for 30 s, 55 °C for 60 s, and 68 °C for 20 s. Gene expression (fold change) was calculated using the 2^(−ΔΔCT)^ method using human β-actin messenger RNA as the housekeeping gene. The β-actin primers used for this analysis were 5′-TGGATCAGCAAGCAGGAGTATG-3′ and 5′-GCATTTGCGGTGGACGAT-3′. The SARS-CoV-2 spike primers were 5′-CAATGGTTTAACAGGCACAGG-3′ and 5′-CTCAAGTGTCTGTGGATCACG-3′. Remdesivir (Medkoo) at 2.5 μM was used as positive control in selected assays.

### Assessment of SARS-CoV-2 protein levels *via* immunofluorescence following drug treatment

Vero CCL-81 cells were grown overnight on coverslips at 1 × 10^5^ cells per well in 12-well plates (Greiner). DMSO or inhibitors were added to the cells in fresh media combined with SARS-CoV-2 virus at a multiplicity of infection of 0.1. After 24 h of incubation, coverslips were collected for staining and imaging of viral antigens. The coverslips were fixed with 4% paraformaldehyde and permeabilized and blocked with a Triton X-100 (0.2%)–bovine serum albumin (3%) solution. Cells were incubated with a 1:250 dilution of mouse monoclonal anti-SARS-CoV/SARS-CoV-2 spike protein antibody (1A9; GeneTex) at room temperature for 1.5 h. Next, they were washed twice and then incubated with Alexa Fluor 647 secondary antimouse antibodies (Invitrogen), diluted at 1:1000, and 4′,6-diamidino-2-phenylindole (1 μg/ml) was added for 1 h at room temperature prior to being washed (two times). Antibodies were diluted in blocking buffer, and PBS containing 0.3% bovine serum albumin was used for the wash steps. Samples were visualized using an Olympus 1 × 81 spinning-disk confocal microscope. Images were analyzed using Volocity (PerkinElmer) or Gen5 (BioTek) software.

### Data analysis and software

GraphPad Prism (GraphPad Software, Inc) was used for general plotting and statistics. Chemical structures were generated using ChemDraw (ChemAxon), and chemical properties (Fig. S19) were predicted using Percepta software (ACD/Labs).

### Quantification and statistical analysis

Numbers of trial replicates and appropriate statistical measures and tests are indicated in the figure captions.

## Data availability

All data relating to this study are included in the article and supporting information files. Inquiries relating to this study and reagent requests should be directed to the corresponding author, Dr Basil P. Hubbard (basil.hubbard@utoronto.ca). All new plasmid constructs generated from this project will be made publicly available on Addgene (“http://www.addgene.org”). Structural data corresponding to Nsp15-H250A have been deposited in PDB (accession code: 8D34). Raw LC–MS/MS data files have been deposited in the MassIVE repository (“https://massive.ucsd.edu”) and are freely available: MSV000092541.

## Supporting information

This article contains supporting information (21, 75, 76).

## Conflict of interest

The authors declare that they have no conflicts of interest with the contents of this article.
