# Supplementary material for: Reversible and irreversible inhibitors of coronavirus Nsp15 endoribonuclease
Source: J Biol Chem. 2023 Oct 11;299(11):105341. doi: 10.1016/j.jbc.2023.105341 (PMC10656235; doi:10.1016/j.jbc.2023.105341)
Supplement: Supporting information [file mmc1.pdf]

Reversible and irreversible inhibitors of coronavirus Nsp15 endoribonuclease

Jerry Chen, Rabih Abou Farraj, Daniel Limonta, Seyed Amir Tabatabaei Dakhili, Evan M. Kerek, Ashim Bhattacharya, Filip M. Reformat, Ola M. Mabrouk, Benjamin Brigant, Tom A. Pfeifer, Mark T. McDermott, John R. Ussher, Tom C. Hobman, J.N. Mark Glover, Basil P. Hubbard

Supporting Information

**Supplemental Figure 1**	Optimization of Nsp15 FRET-based activity assay
**Supplemental Figure 2**	Summaries relating to the primary and secondary inhibitor screens
**Supplemental Figure 3**	Validation of putative Nsp15 inhibitors using multi-substrate FRET-based assays
**Supplemental Figure 4**	Amplex Red assay of lead hits
**Supplemental Figure 5**	Validation of lead hits using a gel-based cleavage assay
**Supplemental Figure 6**	Effect of detergents on the inhibitory capacity of the lead hits
**Supplemental Figure 7**	Nsp15 dispersity in the presence of compounds
**Supplemental Figure 8**	Non-specific compound-RNA interactions evaluated by fluorescence polarization
**Supplemental Figure 9**	Effect of inhibitors on Michaelis-Menten kinetics
**Supplemental Figure 10**	Effect of dithiothreitol (DTT) on compound inhibition
**Supplemental Figure 11**	MALDI-TOF mass spectrometry following treatment of Nsp15 with inhibitors
**Supplemental Figure 12**	Size exclusion chromatography analysis of C291/C293 mutant proteins
**Supplemental Figure 13**	Crystal structure of Nsp15-H250A
**Supplemental Figure 14**	Proposed binding of inhibitors in both catalytic and allosteric sites
**Supplemental Figure 15**	Determination of lead compound CC_{50} values in Vero CCL-81 cells
**Supplemental Figure 16**	Visualization of SARS-CoV-2 infection levels in response to Nsp15 inhibitors
**Supplemental Figure 17**	Alignment of Nsp15 protein sequence in several related coronaviruses.
**Supplemental Figure 18**	Effects of lead hits on SARS-CoV-2 Nsp15 homologs and related enzymes
**Supplemental Figure 19**	Chemical properties of lead inhibitors
**Supplemental Table 1**	Sequences of *E. coli* optimized Nsp15s
**Supplemental Table 2**	Primers used for Nsp15 cloning and sequencing
**Supplemental Table 3**	RNA substrate sequences for Nsp15 FRET-based Assay

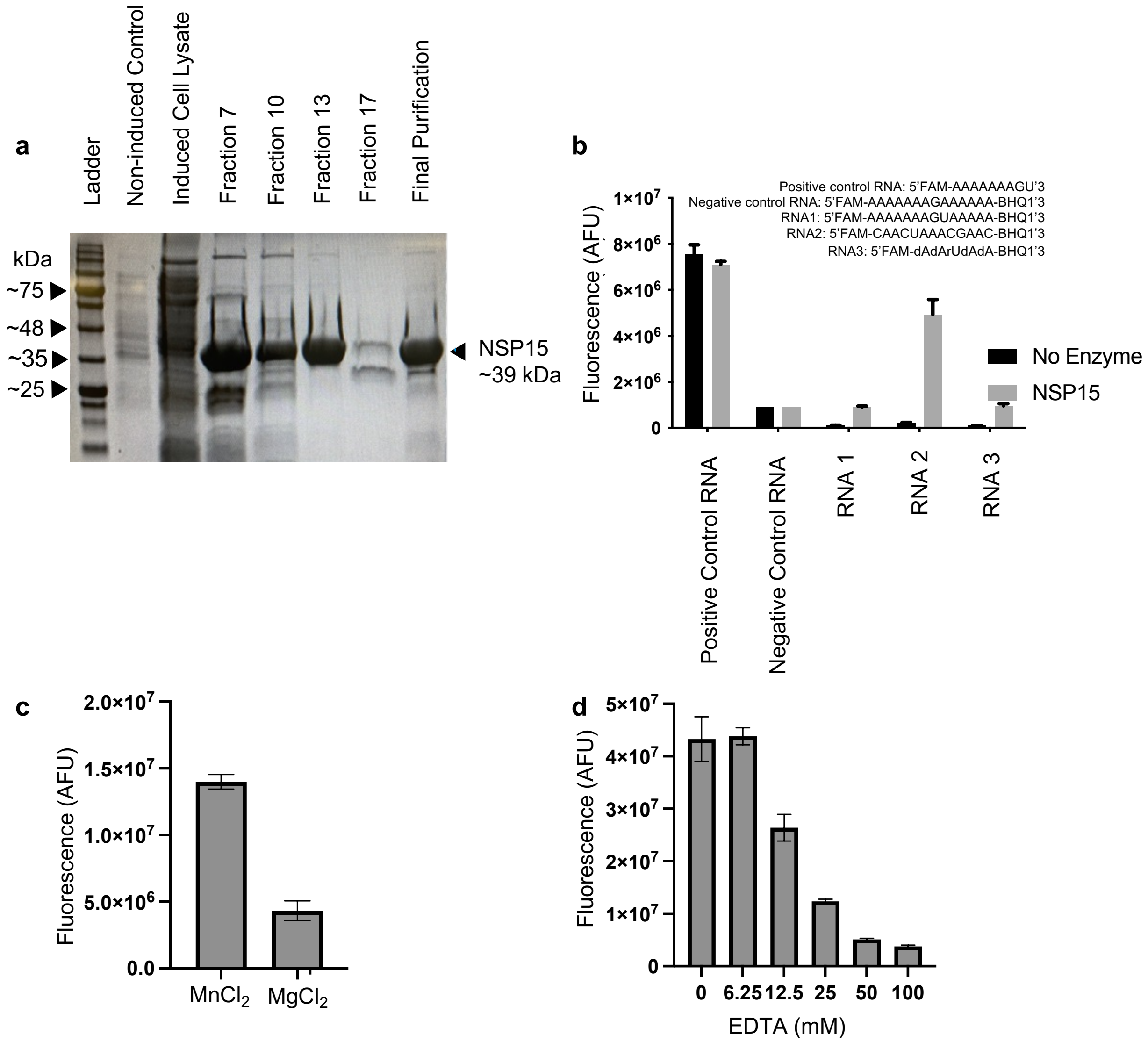

a
Ladder
Non-induced Control
Induced Cell Lysate
Fraction 7
Fraction 10
Fraction 13
Fraction 17
Final Purification
kDa
~75
~48
~35
~25
NSP15
~39 kDa
b
Positive control RNA: 5'FAM-AAAAAAAGU'3
Negative control RNA: 5'FAM-AAAAAAAGAAAAAA-BHQ1'3
RNA1: 5'FAM-AAAAAAAGUAAAAAA-BHQ1'3
RNA2: 5'FAM-CAACUAAACGAAC-BHQ1'3
RNA3: 5'FAM-dAdArUdAdA-BHQ1'3
Fluorescence (AFU)
1×10^7
8×10^6
6×10^6
4×10^6
2×10^6
0
No Enzyme
NSP15
Positive Control RNA
Negative Control RNA
RNA 1
RNA 2
RNA 3
c
Fluorescence (AFU)
2.0×10^7
1.5×10^7
1.0×10^7
5.0×10^6
0.0
MnCl2
MgCl2
d
Fluorescence (AFU)
5×10^7
4×10^7
3×10^7
2×10^7
1×10^7
0
0
6.25
12.5
25
50
100
EDTA (mM)

Figure S1. Optimization of Nsp15 FRET-based activity assay. a) Coomassie stained gel showing bands corresponding to purified recombinant Nsp15 protein. **b)** Graph showing the results of a FRET-based activity assay comparing Nsp15 cleavage activity against various RNA reporter substrates. Reactions were allowed to proceed for 60 mins. An RNA lacking a quencher was used as a positive control, and an RNA without any uracil bases was used as a negative control; Mean + SD is shown (n = 3). **c)** Graph comparing Nsp15 cleavage activity using the RNA2 substrate in buffer containing either 5mM Mn^{2+} or 5mM Mg^{2+} following 40 minutes of incubation; Mean ± SD is shown (n = 3). **d)** Graph showing the results of a titration of EDTA (pH 8.0) concentrations versus Nsp15 activity. The concentration of RNA used was 1 μM, and reactions were allowed to proceed for 40 mins; Mean ± SD is shown (n = 3). All experiments were repeated three times with similar results.

a

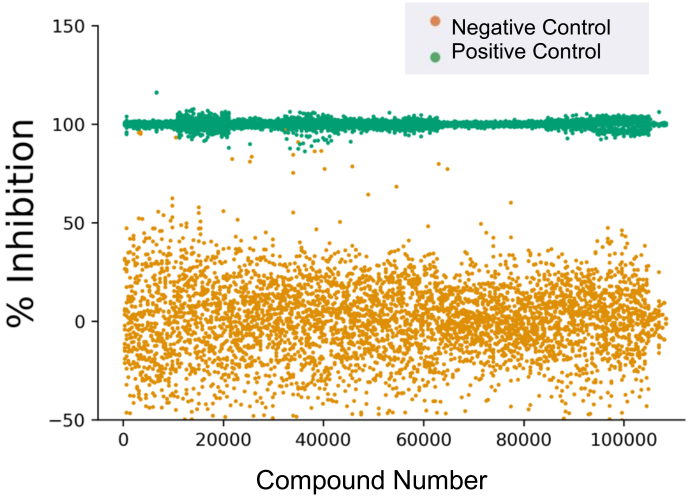

b

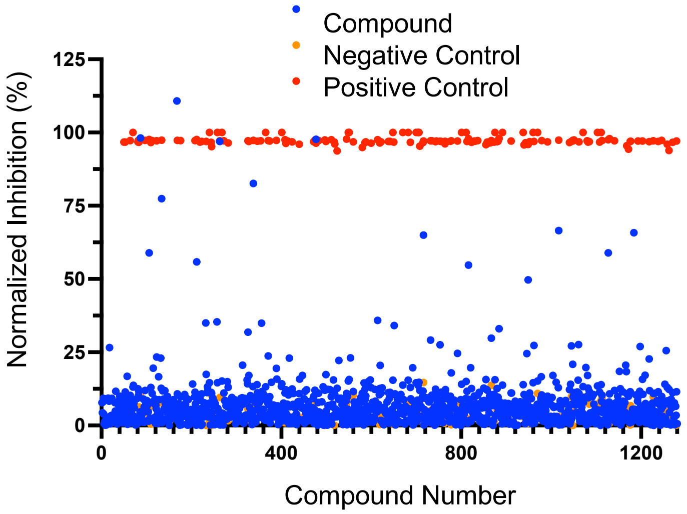

c

	# Compounds
Total (secondary screen)	1280
> 2σ	40
> 3σ	23

Figure S2. Summaries relating to primary and secondary inhibitor screens. a) Plot showing the distribution of negative controls included in the primary high-throughput screen using the FAM-BHQ1 RNA substrate. **b)** Graphic plot summarizing the results of the secondary screen using the Cy5-BHQ2 RNA substrate, encompassing the top 1280 compounds that displayed fluorescence values greater than two standard deviations from the mean in the primary screen. Inhibition % was normalized using control reactions in the absence of enzyme. Negative control denotes enzymatic reactions carried out in the presence of DMSO only. **c)** Table summarizing the results from the secondary screen. Mean and standard deviations were calculated independently for each of 4 plates screened, and hits above 2σ and 3σ on each plate were noted. The total number of hits meeting these criteria are indicated in the table.

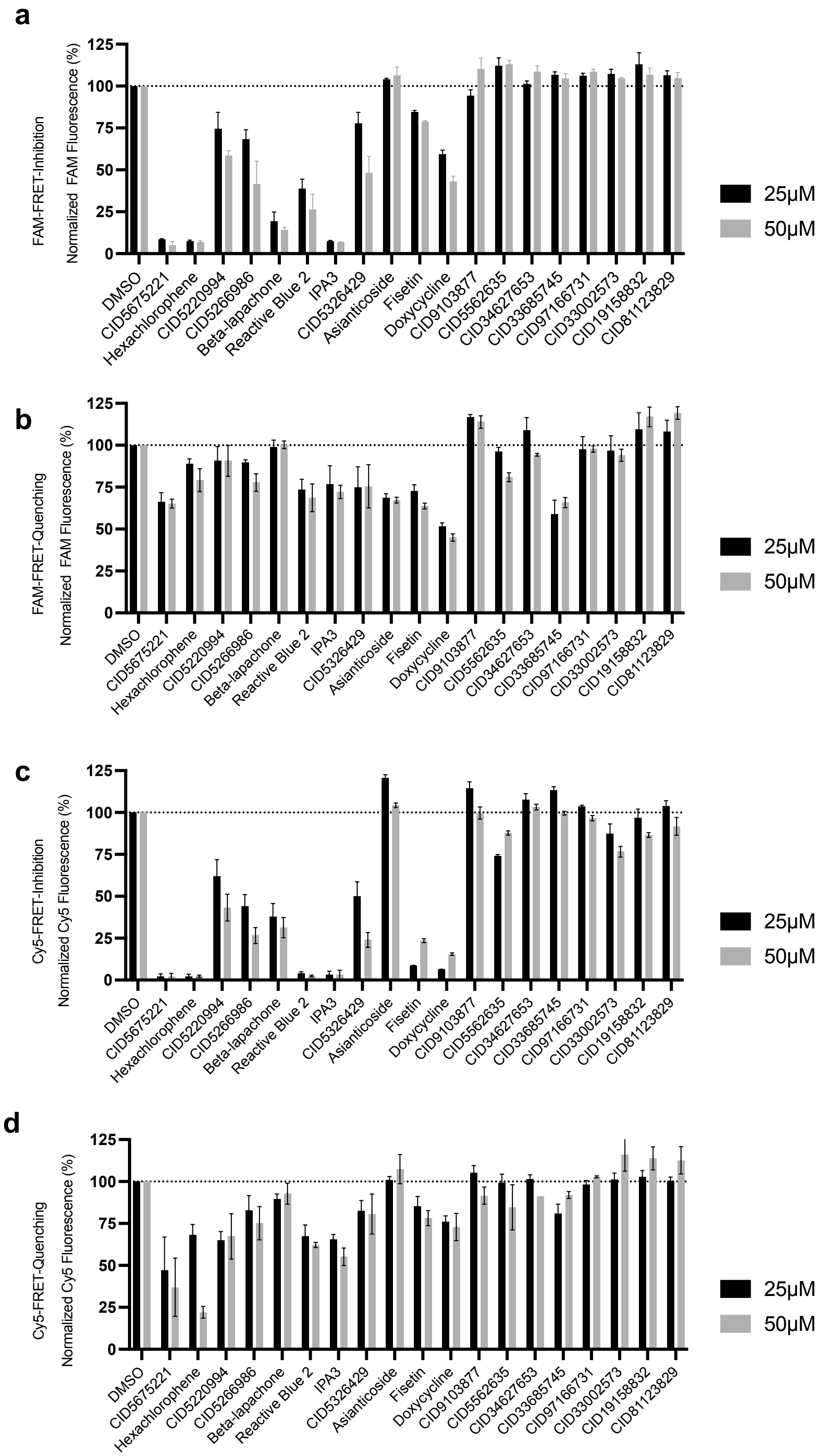
a
b
c
d
FAM-FRET-Inhibition
FAM-FRET-Quenching
Cy5-FRET-Inhibition
Cy5-FRET-Quenching
Normalized FAM Fluorescence (%)
Normalized Cy5 Fluorescence (%)
0
25
50
75
100
125
DMSO
CID5675221
Hexachlorophene
CID5220994
CID5266986
Beta-lapachone
Reactive Blue 2
IPA3
CID5326429
Asianticoside
Fisetin
Doxycycline
CID9103877
CID5562635
CID34627653
CID33685745
CID97166731
CID33002573
CID19158832
CID81123829
25μM
50μM

Figure S3. Validation of putative Nsp15 inhibitors using multi-substrate FRET-based assays. Evaluation of lead compounds in FRET assays using **a)** RNA2 FAM-BHQ1, **b)** Positive Control FAM, **c)** RNA2 Cy5-BHQ2, and **d)** Positive Control Cy5 substrates. Signals were normalized to DMSO control samples. Measurements taken in the presence of the Positive Control substrates were reflective of the ability of the compounds to quench the indicated fluorescent signal. Nsp15 and RNA concentrations were 1 ng/μl and 1 μM, respectively. Compounds were tested at 25 μM and 50 μM, and reactions were allowed to proceed for ~12 mins. Mean ± SD is shown (n = 3)**.** All experiments were repeated three times with similar results.

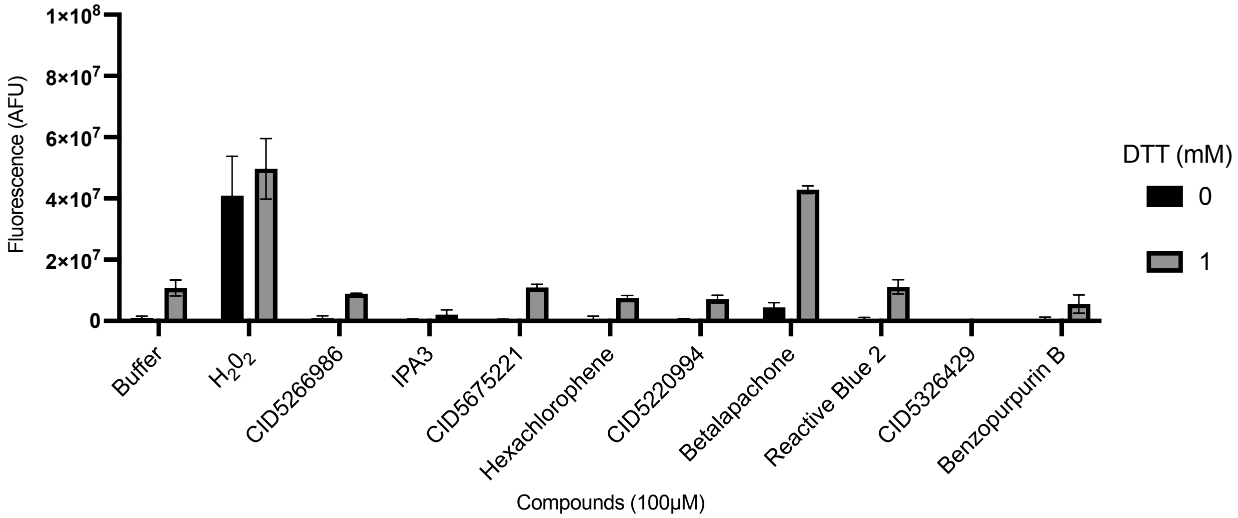

Figure S4. Amplex Red assay of lead hits. Graph displaying results of an Amplex red assay performed using the indicated compounds, in the absence or presence of 1 mM DTT. Reactions were allowed to proceed for 15 minutes, and 10 µM of H_20_2 was used as a positive control; Mean ± SD is shown (n = 3).

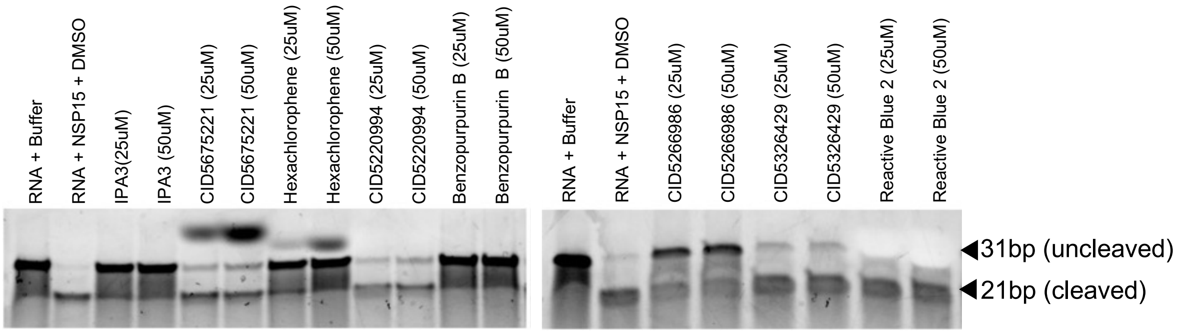

Figure S5. Validation of lead hits using a gel-based cleavage assay.

Representative gels showing the results of a Nsp15 native RNA cleavage assay. Compounds were tested at 25 μM or 50 μM. Reactions were allowed to proceed for 1 hr at 37 ºC. Benzopurpurin B was used as a positive control. Experiments were repeated three times with similar results. Note that inhibition by CID5326429 was sporadic, and not consistent amongst the trials; therefore, this compound was de-prioritized.

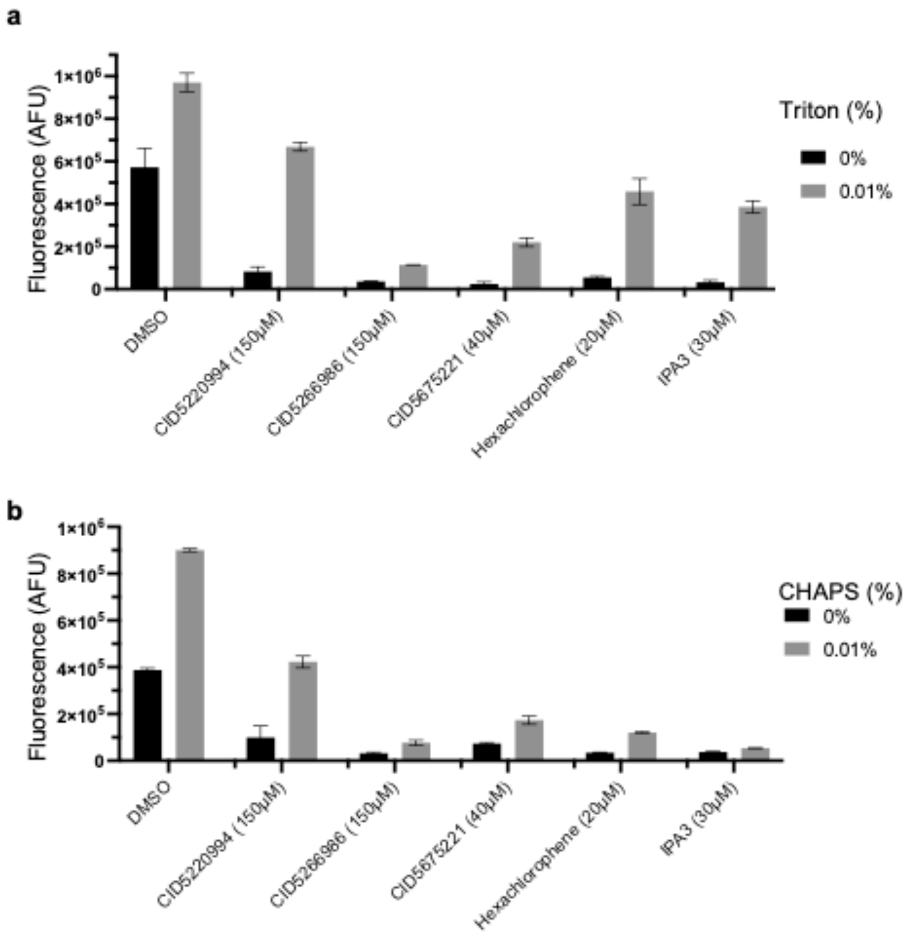

Figure S6. Effect of detergents on the inhibitory capacity of the lead hits. Effect of the addition of **a)** 0.01% Triton-X100 or **b)** 0.01% CHAPS to the reaction buffer. Nsp15 (1 ng/µl) was incubated with RNA2 (1 µM) with the indicated compounds, in the absence or presence of the detergent. Reactions were allowed to proceed for ~12 mins. Mean ± SD is shown (n = 3). All experiments were repeated two times with similar results.

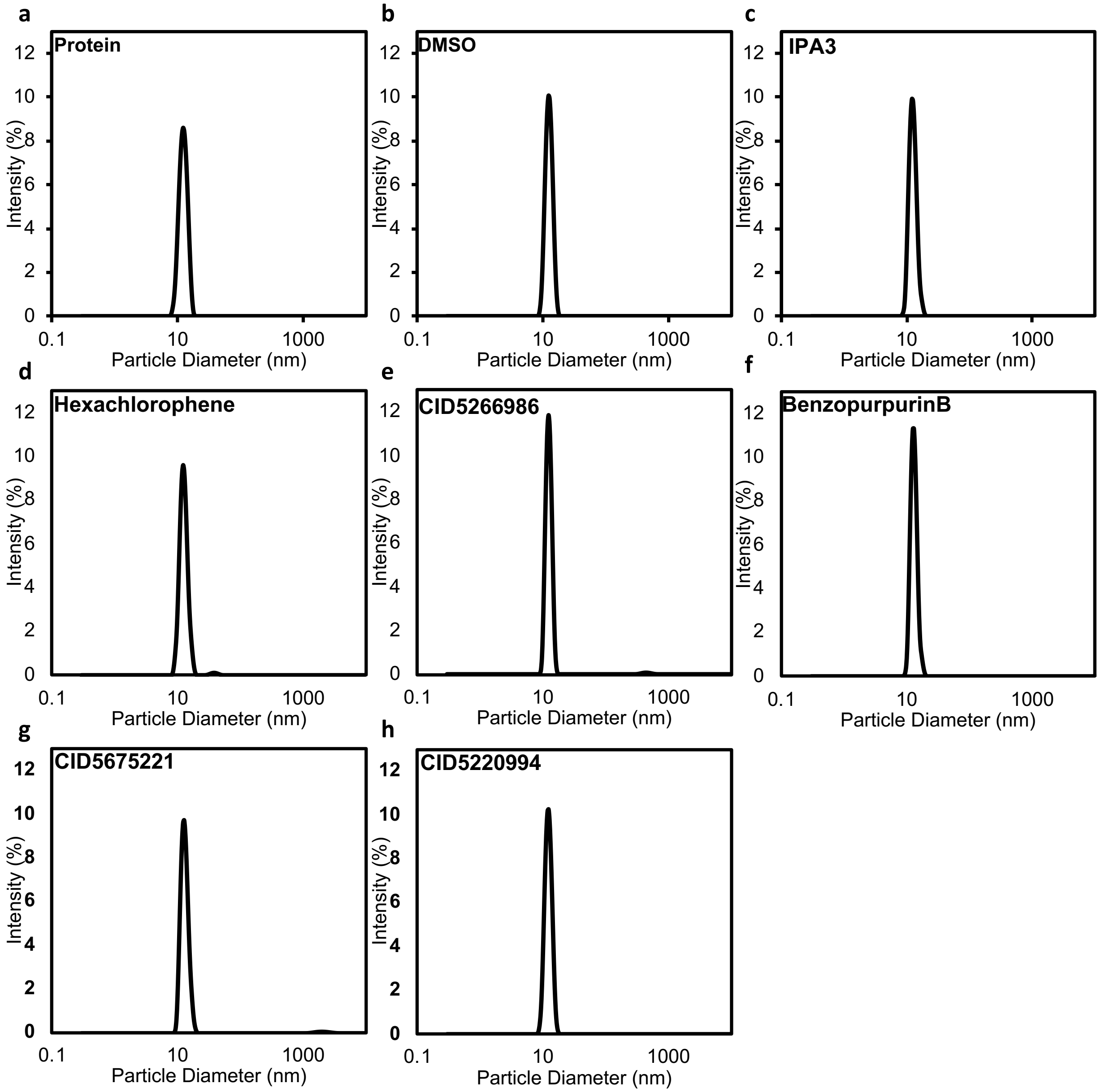

Figure S7. Nsp15 dispersity in the presence of compounds. Intensity size distribution (particle diameter in nm) of **a)** Nsp15 protein in the absence of any compound, or Nsp15 protein in the presence of **b)** DMSO, **c)** IPA-3, **d)** Hexachlorophene **e)** CID5266986 **f)** Benzopurpurin B (control compound), **g)** CID5675221, and **h)** CID5220994. Plots were fit to a Gaussian distribution. Experiments were repeated twice with similar results.

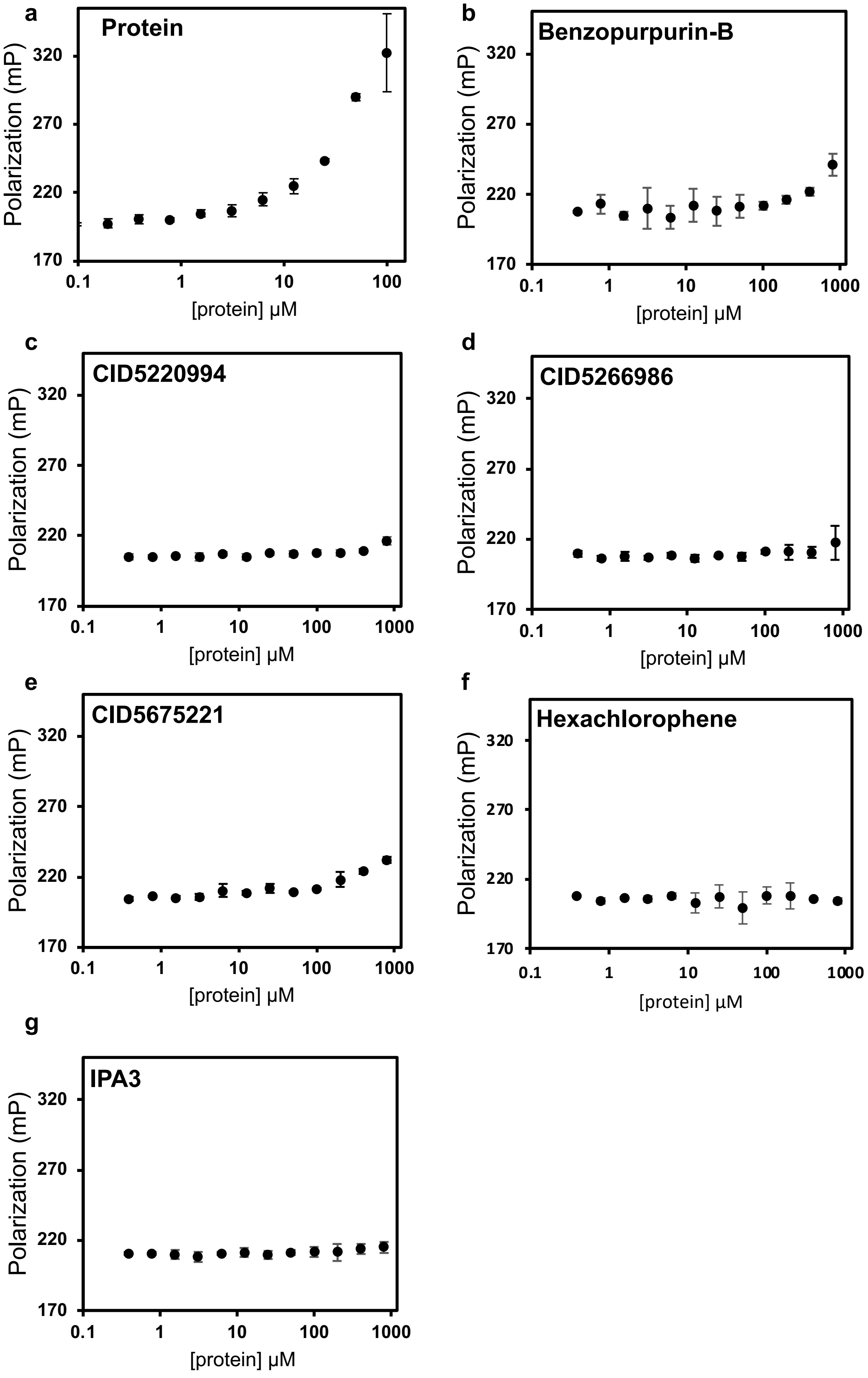
a
Protein
b
Benzopurpurin-B
c
CID5220994
d
CID5266986
e
CID5675221
f
Hexachlorophene
g
IPA3
Polarization (mP)
170
220
270
320
0.1
1
10
100
1000
[protein] μM

Figure S8. Non-specific compound-RNA interactions evaluated by fluorescence polarization. **a)** Polarization measurements from a titration of 0.2 μM to 100 μM of Nsp15 protein to 20 nM of fluorescein-labelled RNA. An accurate dissociation constant (K_d) could not be determined because a saturation point was not achieved. Titrations of 0.4 μM to 800 μM of **b)** Benzopurpurin B, **c)** CID5220994, **d)** CID5266986, **e)** CID5675221, **f)** Hexachlorophene, and **g)** IPA-3 to 20 nM of fluorescein-labelled RNA. For each data point, Mean ± SD is shown (n = 3).

a

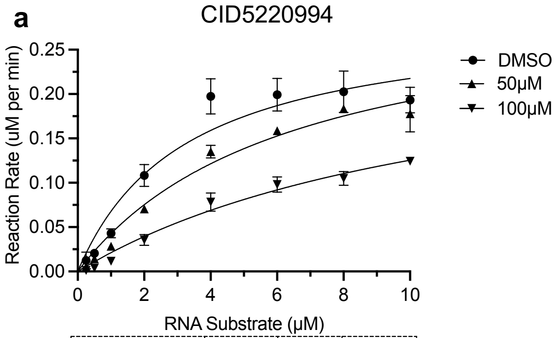

	DMSO	50μM	100μM
Best-fit values			
Vmax	0.29	0.31	0.28
Km	3.3	6.4	12
Std. Error			
Vmax	0.028	0.030	0.047
Km	0.83	1.2	3.3

b

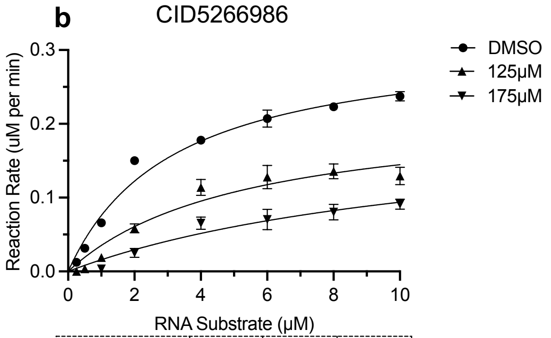

	DMSO	125μM	175μM
Best-fit values			
Vmax	0.32	0.22	0.21
Km	3.2	5.3	12
Std. Error			
Vmax	0.016	0.029	0.058
Km	0.41	1.5	5.3

c

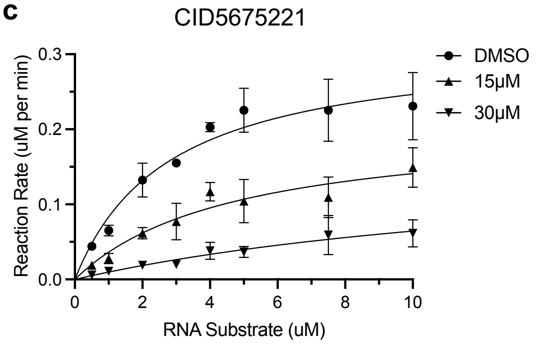

	DMSO	15μM	30μM
Best-fit values			
Vmax	0.32	0.21	0.17
Km	2.8	4.7	16
Std. Error			
Vmax	0.027	0.034	0.087
Km	0.62	1.6	12

d

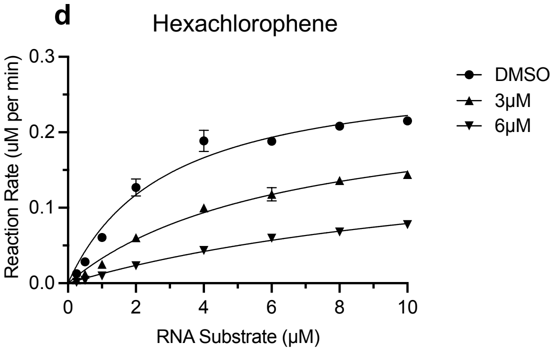

	DMSO	3μM	6μM
Best-fit values			
Vmax	0.29	0.24	0.19
Km	2.9	6.3	14
Std. Error			
Vmax	0.014	0.014	0.021
Km	0.39	0.75	2.3

e

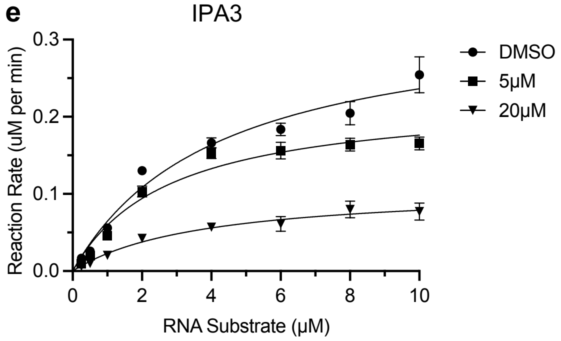

	DMSO	5μM	20μM
Best-fit values			
Vmax	0.34	0.23	0.11
Km	4.2	2.8	3.8
Std. Error			
Vmax	0.024	0.013	0.0092
Km	0.72	0.47	0.75

f

Compound	K_m diff.	K_m Statistics F=(DFn, DFd)	V_{max} diff.	V_{max} Statistics F=(DFn, DFd)	Predicted Type of inhibition	Computed Ki ± SE
CID5220994 (100 μM)	Yes	P<0.0001 25.93 (1, 22)	No	P=0.4587 F=0.5689 (1, 22)	Competitive	72.9 ± 11.6
CID5266986 (175 μM)	Yes	P<0.0001 23.69 (1, 22)	Yes	P=0.0397 4.782 (1, 22)	Mixed	64.9 ± 16.9
CID5675221 (30 μM)	Yes	P=0.0004 F=17.28 (1, 22)	Yes	P<0.0001 F 79.16 (1, 22)	Mixed	6.71 ± 2.31
Hexachlorophene (6 μM)	Yes	P<0.0001 F=65.12 (1, 22)	Yes	P=0.0280 F=5.537 (1, 22)	Mixed	1.23 ± 0.30
IPA3 (20 μM)	No	P=0.9059 F=0.01431 (1, 22)	Yes	P=0.0003 F= 18.01 (1, 22)	Non-competitive	10.81 ± 0.87

Figure S9. Effect of inhibitors on Michaelis-Menten kinetics. Reaction rate versus a titration of RNA substrate concentrations ranging from 0 to 10 µM in the absence or presence of the indicated doses of **a)** CID5220994, **b)** CID5266986, **c)** CID5675221, **d)** Hexachlorophene, or **e)** IPA-3. Nsp15 was used at a concentration of 1 ng/µl with the RNA2 substrate, and reactions were allowed to proceed for ~6 minutes at 37ºC. Reaction rate was calculated by converting raw fluorescence values using a standard curve produced using the Positive Control FAM RNA, and dividing by the elapsed time. Plots were fitted to Michaelis-Menten curves ($v=V_{max}[S]/(K_M + [S])$), and the corresponding kinetic parameters were calculated using GraphPad Prism. Mean ± SD for replicates is shown (n = 3), and experiments were repeated three times with similar results. **f)** Table indicating the apparent mechanisms of the various inhibitors with corresponding statistics, and calculated K_i values. Statistics were computed using an extra sum-of-squares F test against average DMSO values taken from 15 trials (K_M of 3.2 µM ± 0.20 and V_{max} of 0.30µM/min ± 0.007), using the mean values of three independent experimental trials. K_i values were computed using the data in **a-e** by fitting to Michaelis-Menten models for competitive (where $K_{MObs}=K_M*(1 + [I]/K_i)$), non-competitive (where $V_{maxObs}=V_{max}/(1 + [I]/K_i)$), and mixed inhibition (where $K_{MObs}=K_M*(1 + [I]/K_i)/(1 + [I]/\alpha K_i)$ and $V_{maxObs}=V_{max}/(1 + [I]/(\alpha K_i))$).

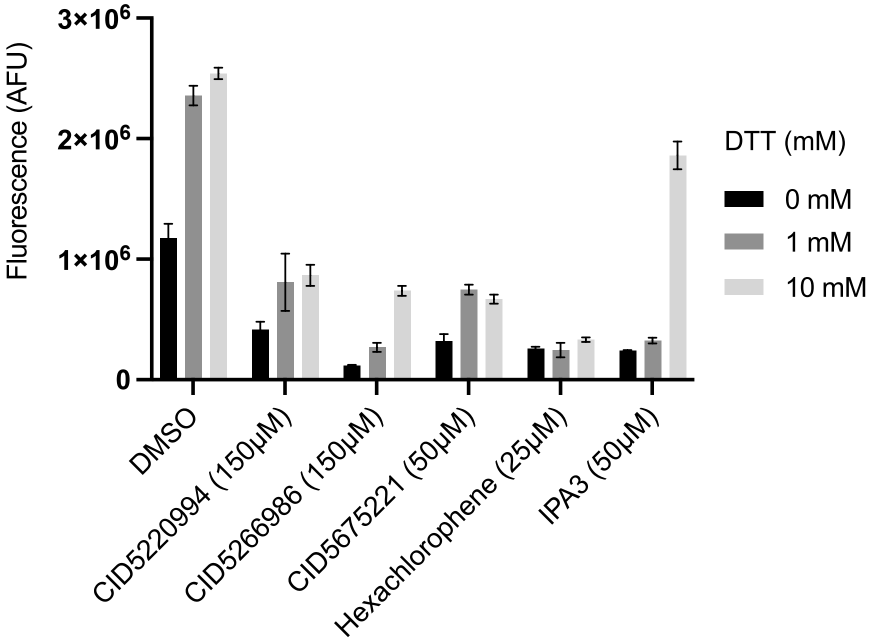

Figure S10. Effect of dithiothreitol (DTT) on compound inhibition. Effects of the indicated concentrations of inhibitors on Nsp15 activity in the absence or presence of 1 mM or 10 mM DTT. Mean ± SD for replicates is shown (n = 3), and experiments were repeated three times with similar results.

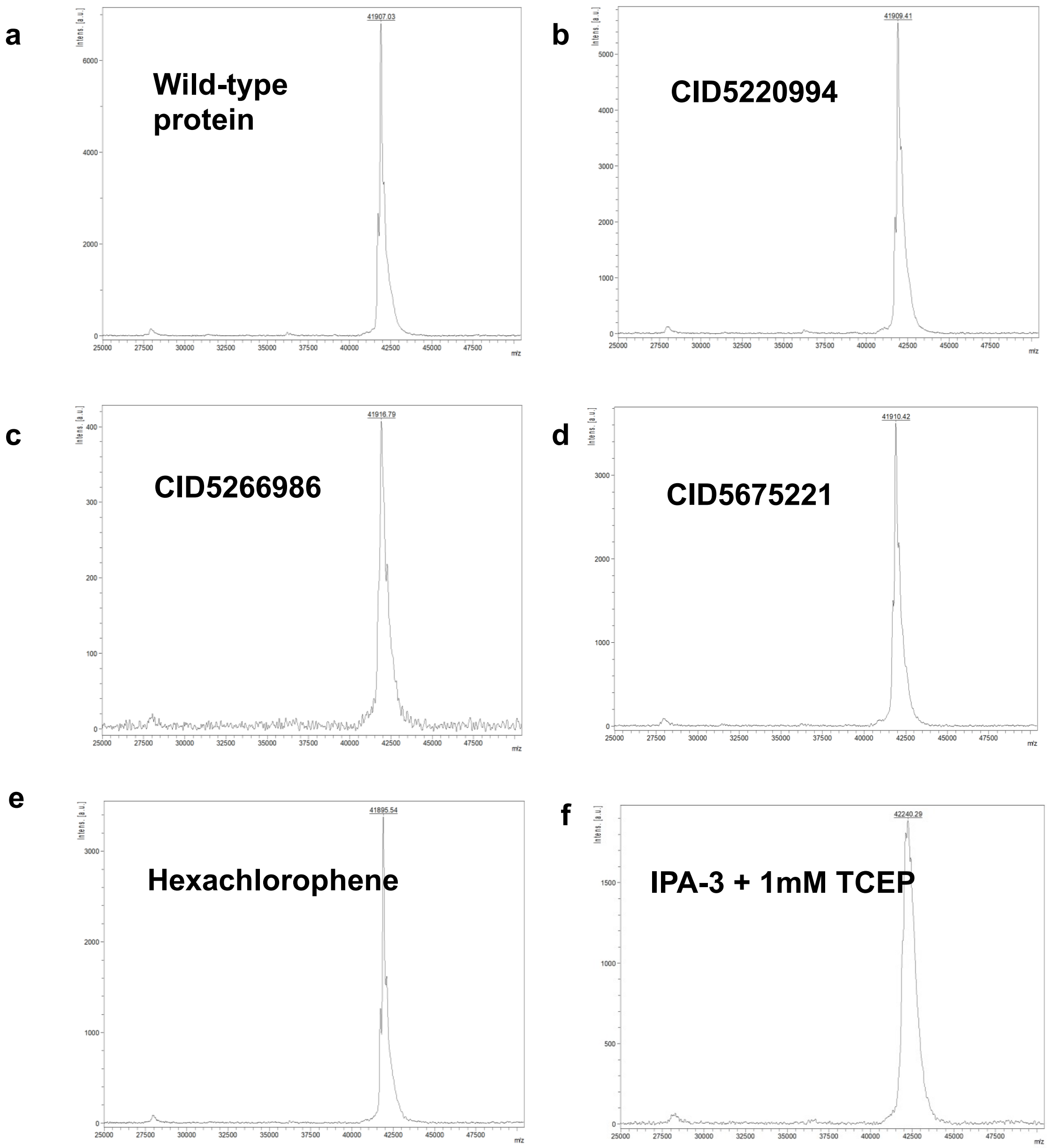
a
Wild-type protein
41907.03
b
CID5220994
41909.41
c
CID5266986
41916.79
d
CID5675221
41910.42
e
Hexachlorophene
41895.54
f
IPA-3 + 1mM TCEP
42240.29

Figure S11. MALDI-TOF mass spectrometry following treatment of Nsp15 with inhibitors. Representative mass spectra of **a)** wild-type Nsp15 protein, and Nsp15 protein following overnight incubation with a 5-fold molar excess of **b)** CID5220994, **c)** CID5266986, **d)** CID5675221, **e)** Hexachlorophene, or **f)** IPA-3. The calculated mass is indicated at the top of each central peak. Experiments were repeated three times, in the absence or presence of 1 mM DTT or 1mM TCEP, with similar results.

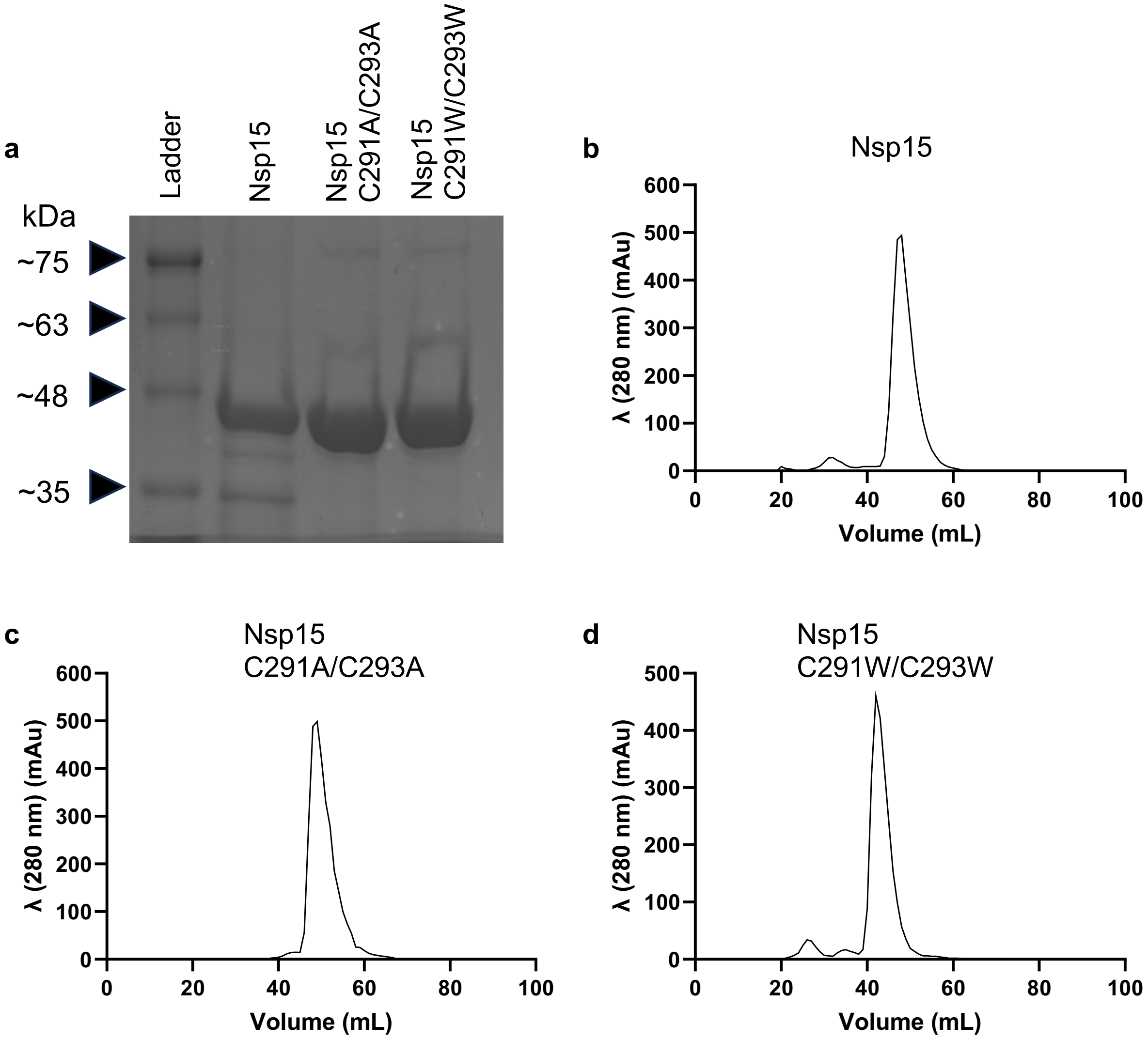
a
Ladder
Nsp15
Nsp15
C291A/C293A
Nsp15
C291W/C293W
kDa
~75
~63
~48
~35
b
Nsp15
λ (280 nm) (mAu)
Volume (mL)
c
Nsp15
C291A/C293A
λ (280 nm) (mAu)
Volume (mL)
d
Nsp15
C291W/C293W
λ (280 nm) (mAu)
Volume (mL)

Figure S12. Size exclusion chromatography analysis of C291/C293 mutant proteins. a) Coomassie stained SDS-PAGE gel showing the relative sizes of purified Wild-type, C291A/C293A, and C291W/C293W mutant Nsp15 proteins. Gel filtration chromatograms showing absorbance versus volume of elution for **b)** Wild-type Nsp15, **c)** Nsp15-C291/C293A, and **d)** Nsp15-C291W/C293W. Samples were analyzed using a Cytiva HiPrep 16/60 Sephacryl S-200 HR column on a Biorad medium pressure liquid chromatography system inside a chamber at 4 °C. The column was equilibrated with 60 mL distilled water followed by 240 mL gel filtration buffer (0.05M sodium phosphate, 0.15M sodium chloride, pH 7.0). The samples were loaded at 0.25 mL/min, and eluted at 0.25 mL/min with gel filtration buffer over 10 hours. A total of 6.5 mg of protein was used in each trial. Experiments were repeated twice with comparable results.

a

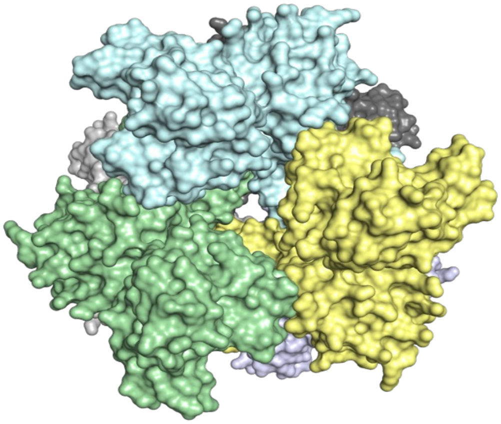

b

**Data Collection**	
Space Group	$P6_3$
Cell Dimensions	
a, b, c (Å)	152.525, 152.525, 109.644
α, β, γ (°)	90, 90, 120
Resolution (Å)	50.0 - 2.91 (2.96-2.910)
R_{meas}	0.270 (1.92)
I/σ (I)	4.3 (1.9)
Completeness (%)	100 (100)
Redundancy	15.900 (11.900)
$CC_{1/2}$	0.64
**Refinement**	
Resolution (Å)	38.13 - 2.910
Reflections used in refinemnet	31298 (2649)
Rwork	0.2462 (0.3170)
Rfree	0.2626 (0.3033)
Number of non-hydrogen atoms	5488
Macromolecules	5488
Ligands	0
Solvent	0
Protein residues	696
Nucleic acid bases	0
B-factor	71.0415
RMS bond length	0.002
RMS bond angles	0.44
Ramachandran favored (%)	97.69
Ramachandran allowed (%)	2.31
Ramachandran outliers (%)	0
Rotamer outliers (%)	0.32
Clashscore	1.82
Number of TLS groups	13

c

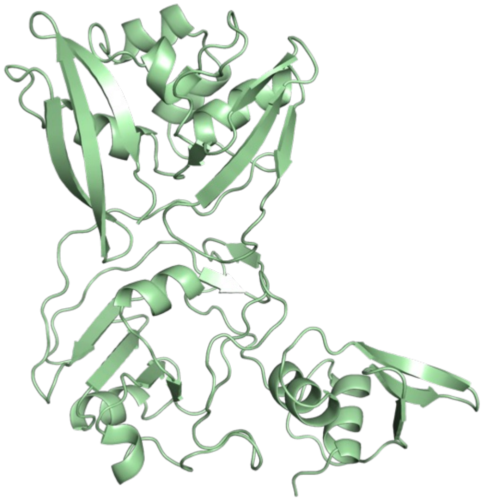

d

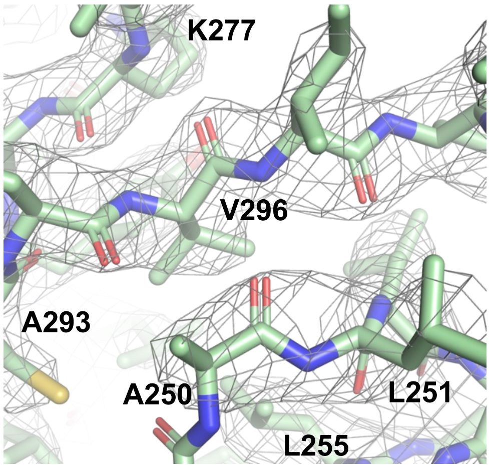

Figure S13. Crystal structure of Nsp15-H250A. a) Surface representation of the hexameric structure of Nsp15-H250A colored with different schemes for each monomer (PDB accession code: 8D34), **b)** Data collection and statistics, **c)** cartoon of the monomeric structure of Nsp15-H250A, and **d)** stick representation of Nsp15-H250A highlighting the density change caused by the active site mutation.

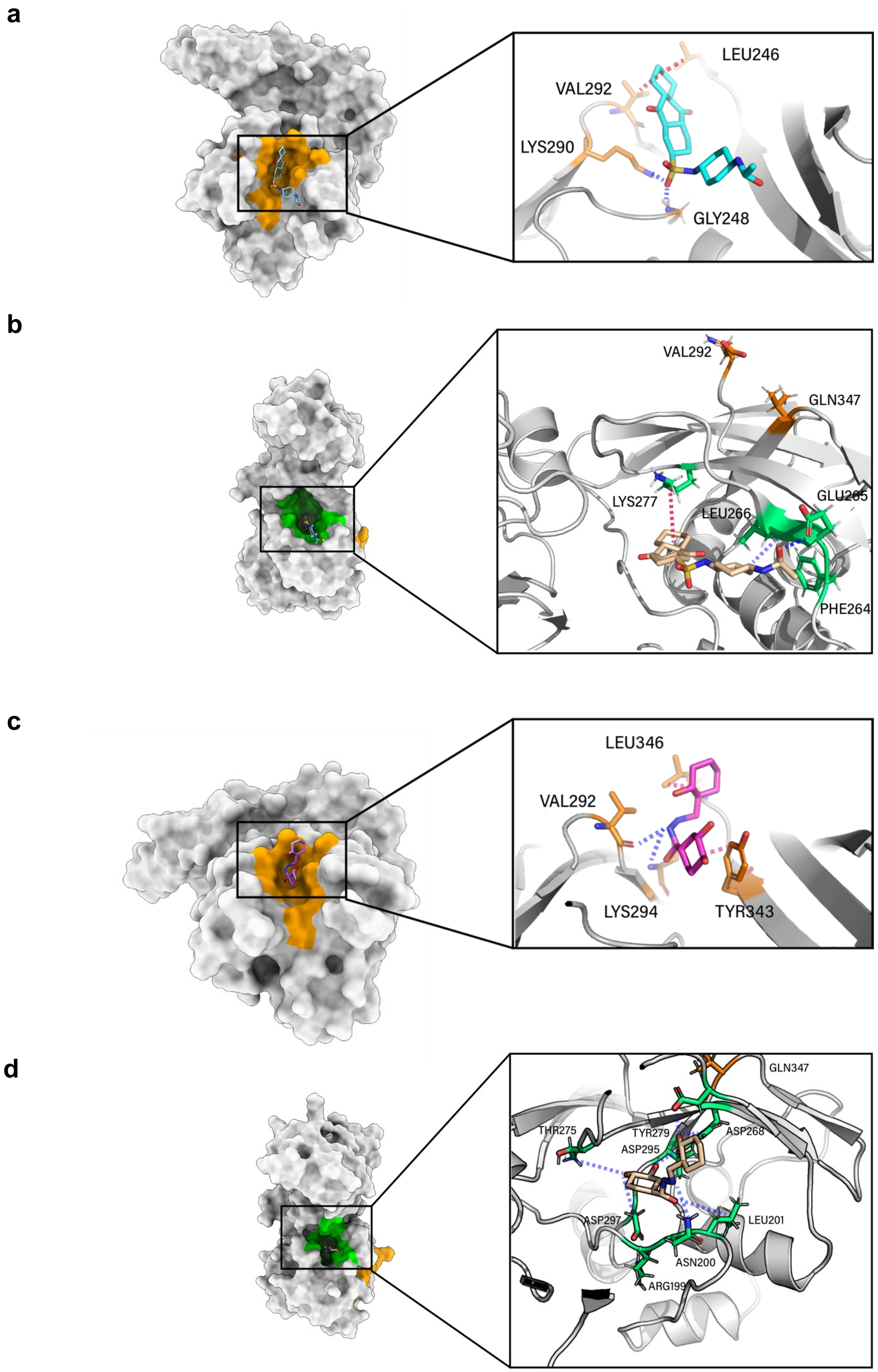
a
LEU246
VAL292
LYS290
GLY248
b
VAL292
GLN347
LYS277
LEU266
GLU265
PHE264
c
LEU346
VAL292
LYS294
TYR343
d
GLN347
THR275
TYR279
ASP268
ASP295
ASP297
LEU201
ASN200
ARG199

Figure S14. Proposed binding of inhibitors in both catalytic and allosteric sites. Proposed binding mode of CID5220994 in the **a)** active site, and **b)** allosteric site of Nsp15. Predicted binding mode of CID5266986 in the **c)** active site, and **d)** allosteric pocket of Nsp15. Hydrogen bonding and hydrophobic interactions are represented using dashed blue and red lines, respectively.

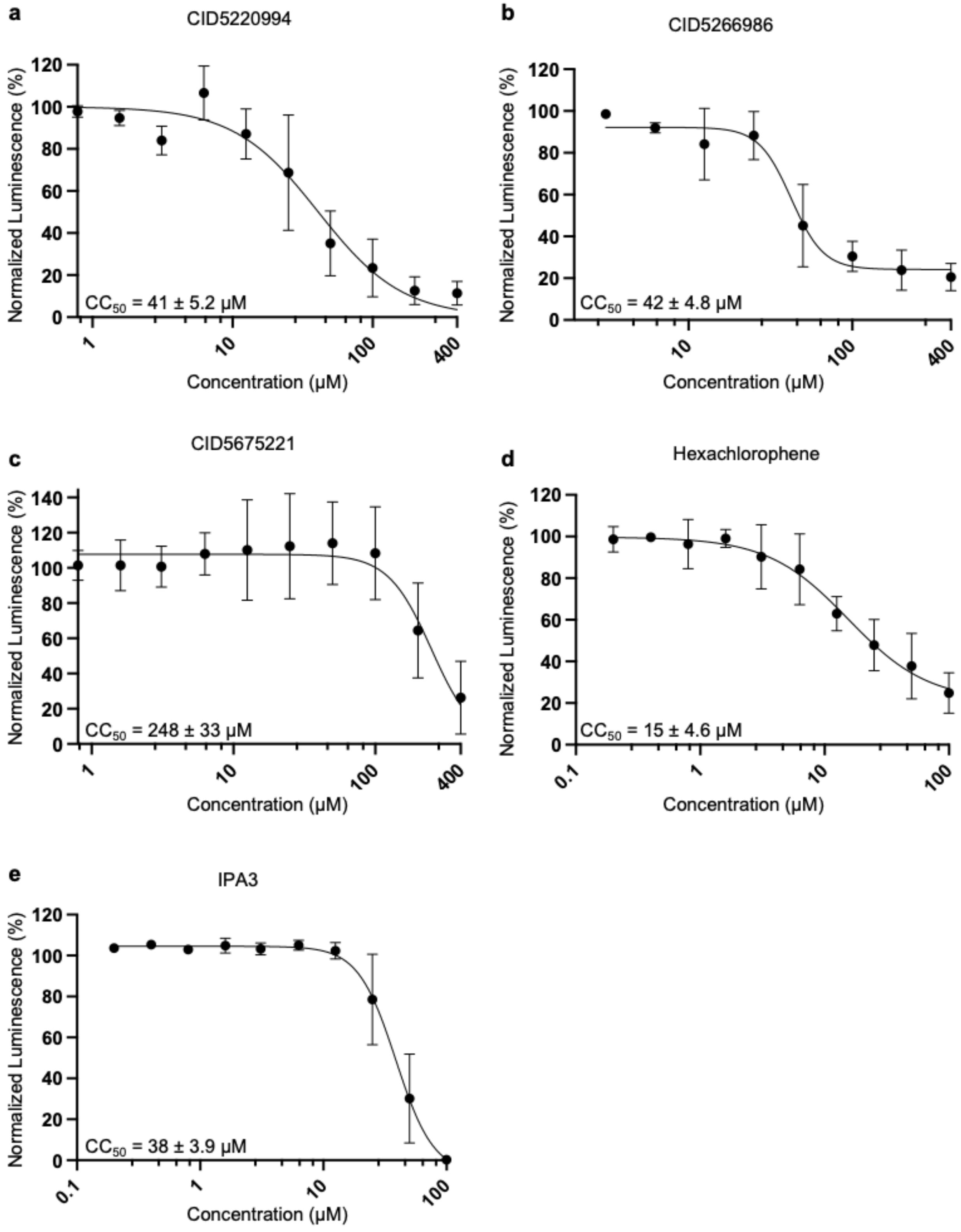

a
CID5220994
Normalized Luminescence (%)
CC50 = 41 ± 5.2 μM
Concentration (μM)
b
CID5266986
Normalized Luminescence (%)
CC50 = 42 ± 4.8 μM
Concentration (μM)
c
CID5675221
Normalized Luminescence (%)
CC50 = 248 ± 33 μM
Concentration (μM)
d
Hexachlorophene
Normalized Luminescence (%)
CC50 = 15 ± 4.6 μM
Concentration (μM)
e
IPA3
Normalized Luminescence (%)
CC50 = 38 ± 3.9 μM
Concentration (μM)

Figure S15. Determination of lead compound CC_{50} values in Vero CCL-81 cells. Plots showing cell viability in the presence of increasing doses of **a)** CID5220994, **b)** CID5266986, **c)** CID5675221, **d)** Hexachlorophene, and **e)** IPA-3. Compounds were incubated with cells for 24 h and viability was assessed using the CellTiterGlo assay. Results were normalized to a DMSO control. Plots were fitted to [inhibitor] vs normalized response curves with variable slope ($y=100/(1+(IC_{50}/[I])^{HillSlope})$) using GraphPad Prism to calculate CC_{50} values; Mean ± SD is shown (n = 3).

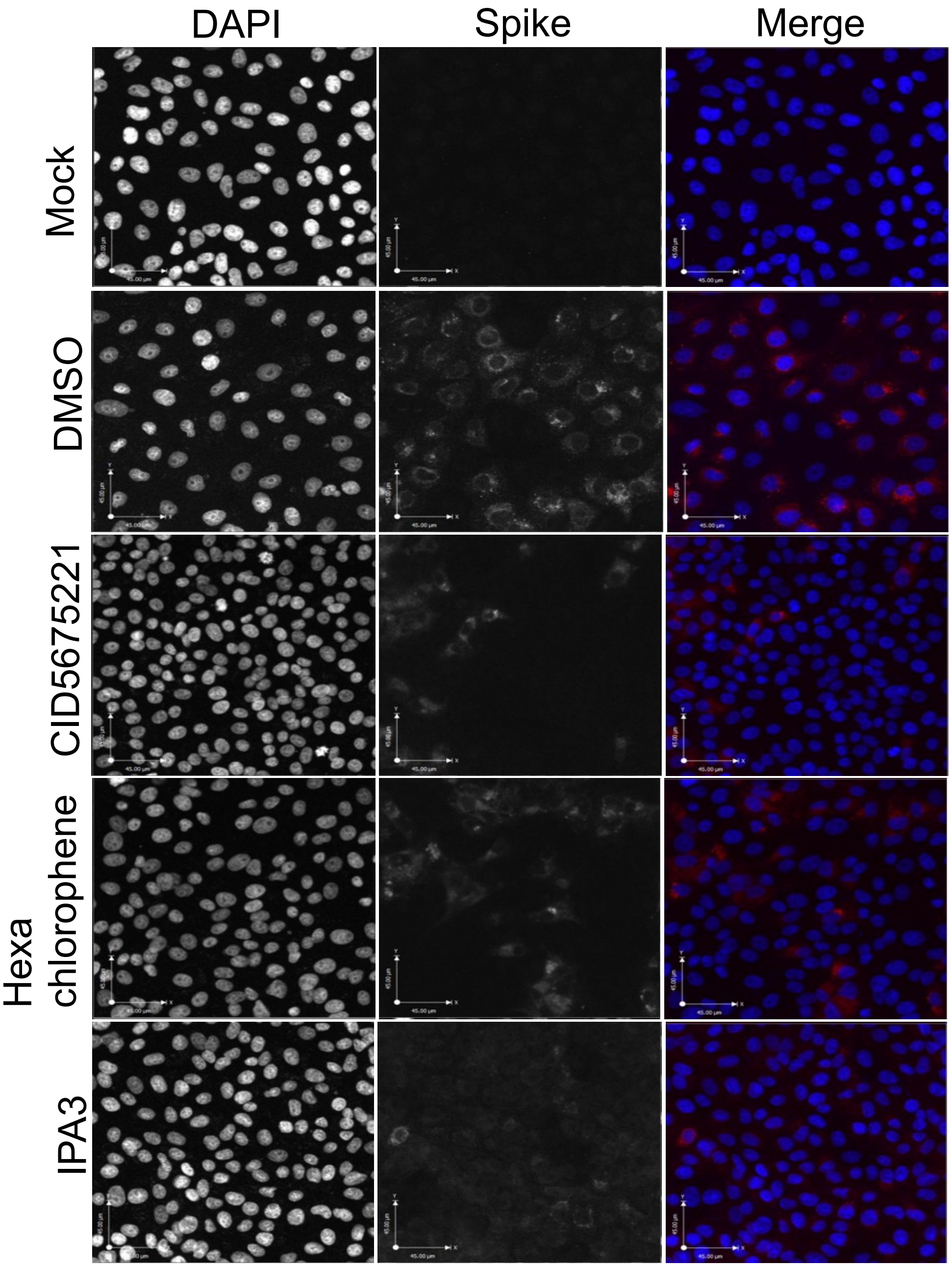
DAPI
Spike
Merge
Mock
DMSO
CID5675221
Hexa chlorophene
IPA3

Figure S16. Visualization of SARS-CoV-2 infection levels in response to Nsp15 inhibitors. Representative confocal images (20X) of Vero CCL-81 cells infected with SARS-CoV-2 at an MOI of 0.1 and treated with sub-toxic concentrations of CID5675221 (50μM), Hexachlorophene (3.1 μM), IPA3 (12.5μM) or DMSO, as indicated, for 24 hours. After fixation, SARS-CoV-2-infected cells were stained using a mouse monoclonal antibody to the coronavirus spike protein; the secondary antibody was anti-mouse Alexa Fluor 647. Nuclei were stained with DAPI. Confocal images were acquired using a spinning disk confocal microscope with Volocity 6.2.1 software.

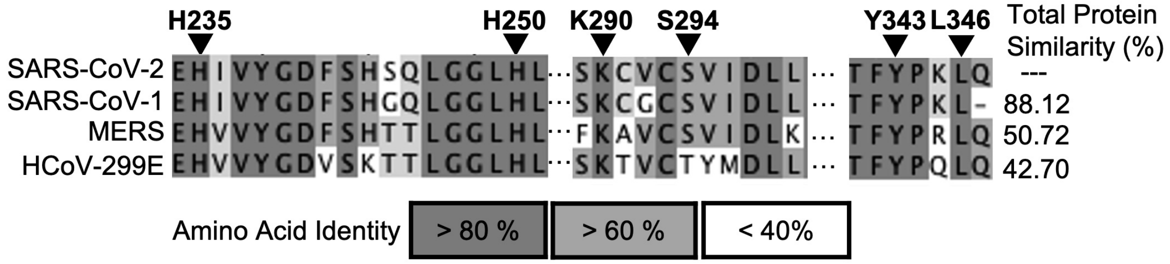

Figure S17. Alignment of Nsp15 protein sequence in several related coronaviruses. Alignment was performed with ClustalW, and coloring was performed using JalView.

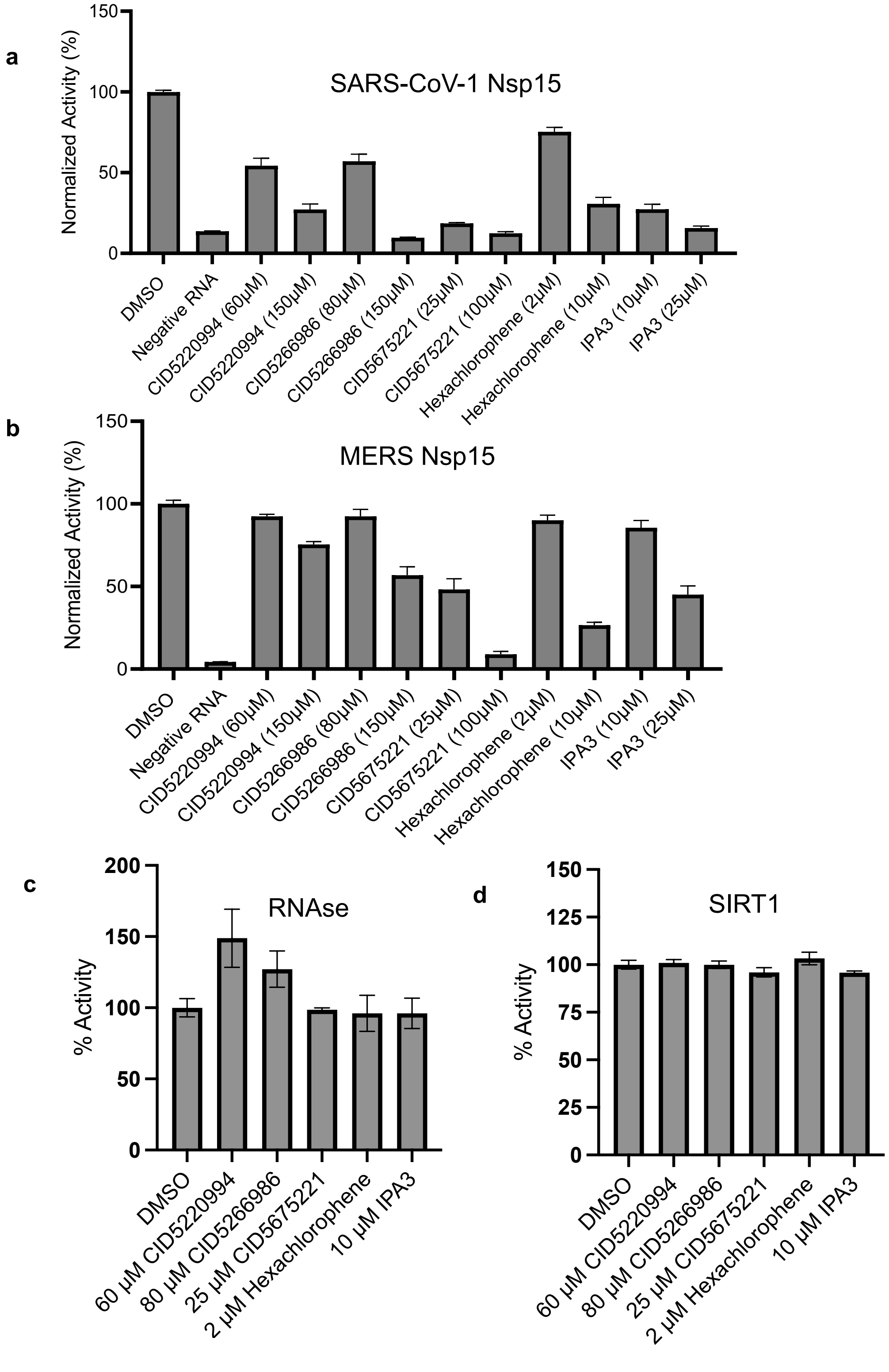
a
SARS-CoV-1 Nsp15
Normalized Activity (%)
150
100
50
0
DMSO
Negative RNA
CID5220994 (60μM)
CID5220994 (150μM)
CID5266986 (80μM)
CID5266986 (150μM)
CID5675221 (25μM)
CID5675221 (100μM)
Hexachlorophene (2μM)
Hexachlorophene (10μM)
IPA3 (10μM)
IPA3 (25μM)
b
MERS Nsp15
Normalized Activity (%)
150
100
50
0
DMSO
Negative RNA
CID5220994 (60μM)
CID5220994 (150μM)
CID5266986 (80μM)
CID5266986 (150μM)
CID5675221 (25μM)
CID5675221 (100μM)
Hexachlorophene (2μM)
Hexachlorophene (10μM)
IPA3 (10μM)
IPA3 (25μM)
c
RNAse
% Activity
200
150
100
50
0
DMSO
60 μM CID5220994
80 μM CID5266986
25 μM CID5675221
2 μM Hexachlorophene
10 μM IPA3
d
SIRT1
% Activity
150
125
100
75
50
25
0
DMSO
60 μM CID5220994
80 μM CID5266986
25 μM CID5675221
2 μM Hexachlorophene
10 μM IPA3

Figure S18. Effects of lead hits on SARS-CoV-2 Nsp15 homologs and unrelated enzymes. Assay of inhibitors at ~IC_{50} and ~IC_{100} concentrations against Nsp15 proteins from **a)** SARS-CoV-1, and **b)** MERS. Nsp15 (1 ng/μl) was incubated with RNA2 (1 μM) with the indicated compounds, and reactions were allowed to proceed for ~15 mins. Mean + SD is shown (n = 3). **c)** Effect of inhibitors on RNAse A activity (45 ng per assay, obtained from NEB), assayed using the same parameters as above. **d)** Effect of inhibitors on SIRT1 activity, measured using the Fleur de Lys assay (Enzo). SIRT1, FdL substrate, and NAD+ were used at concentrations of 200 nM, 25 μM, and 5 mM, respectively. Inhibitors were preincubated with SIRT1 for 30 minutes, and reactions were allowed to proceed for 30 minutes at 37 ^{o}C following addition of substrate. The reactions were terminated by addition of developer reagent and incubated for 15 minutes at room temperature in the dark before being measured on a spectrophotometer. For **c)** and **d)**, results are expressed as a percent activity relative to the DMSO control, and were normalized based on quenching effects of the compounds in the respective assays using control substrates. Mean ± SD is shown (n = 3 independent experiments).

	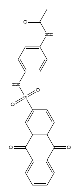	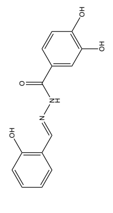	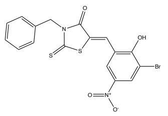	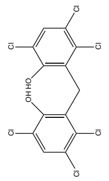	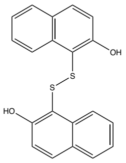
Name	CID5220994	CID5266986	CID5675221	Hexachlorophene	IPA3
Molecular Weight	420.44	272.26	451.32	406.90	350.46
Density	1.49	1.34	1.80	1.71	1.46
# H-bond Donors	2	3	1	2	2
# H-bond Acceptors	7	5	6	2	2
# Aromatic Rings	3	2	2	2	4
LogS (pH = 7.30)	-5.04	-2.87	-2.06	-4.87	-6.06
LogP	2.80	2.47	4.01	7.25	4.92
LogD (pH = 7.30)	2.66	2.34	0.99	6.05	4.70
Strongest pKa (Acid)	7.7 ± 0.2	8.3 ± 0.2	3.7 ± 0.5	6.5 ± 0.5	7.5 ± 0.5

Figure S19. Chemical properties of lead inhibitors. Table of molecular properties for the indicated compounds. Parameters were generated by inputting SMILES notation corresponding to each compound into ACD Labs Percepta software.

Supplemental Table 1: Sequences of *E coli*. optimized Nsp15s. The IDT codon optimization tool was used to optimize the sequences for *Escherichia coli*. Restriction sites (where applicable) are shown in bold.

SARS-CoV-2 Nsp15

5’-
ATGGGCAGCAGCCATCATCATCATCATCACAGCAGCGGCGTGGATCTTGGTACGGAAAA
TCTGTACTTTCAATCCAATGCCATGTCGTTAGAGAACGTAGCTTTTAACGTGGTGAACAA
AGGCCATTTTGATGGTCAACAGGGAGAAGTTCCAGTGTCCATTATAAACAATACGGTATA
CACCAAAGTGGACGGGGTAGATGTAGAACTGTTTGAGAATAAGACGACTTTACCAGTGA
ATGTAGCTTTCGAGTTGTGGGCTAAGAGAAACATCAAGCCAGTACCCGAAGTTAAGATTT
TAAATAATCTTGGGGTAGACATTGCTGCAAATACTGTCATCTGGGACTATAAACGGGACG
CGCCCGCACATATCTCAACTATCGGCGTTTGCAGTATGACAGATATAGCGAAAAAGCCG
ACGGAAACCATATGTGCACCCTTGACCGTTTTCTTCGATGGTCGGGTGGATGGTCAGGT
CGATTTATTCAGAAATGCTCGTAATGGAGTCCTGATTACTGAGGGGAGTGTAAAAGGATT
ACAACCATCAGTGGGACCGAAACAGGCTTCCCTTAATGGTGTTACGCTTATCGGTGAGG
CGGTAAAGACACAGTTCAATTATTACAAAAAGGTGGACGGCGTCGTACAACAGTTACCTG
AAACTTACTTTACGCAGTCGAGAAACTTGCAGGAATTCAAGCCGCGCTCCCAGATGGAA
ATTGATTTTCTGGAGCTGGCTATGGACGAATTTATCGAAAGATATAAGCTGGAAGGATAT
GCTTTCGAACATATAGTATACGGGGATTTTTCTCATTCGCAACTGGGTGGCCTTCATCTT
TTAATAGGCTTAGCGAAGCGTTTCAAGGAATCTCCTTTTGAGTTAGAAGACTTTATTCCGA
TGGACTCCACCGTTAAGAACTATTTCATTACAGATGCACAGACGGGTTCTAGCAAATGTG
TATGCTCTGTAATTGACCTGTTGTTAGACGACTTTGTCGAAATTATCAAAAGCCAGGACC
TGTCCGTAGTCTCGAAGGTTGTGAAAGTGACCATCGACTACACAGAGATATCCTTTATGC
TTTGGTGCAAGGATGGTCACGTTGAAACATTTTATCCCAAGCTGCAGTAA-’3

SARS-CoV-1 Nsp15

5’-
GCCG**GGATCC**AGCCTGGAAAATGTTGCCTATAACGTGGTGAATAAAGGCCATTTTGATG
GTCATGCCGGTGAAGCACCGGTTAGCATTATTAACAATGCCGTTTATACCAAAGTGGATG
GCATTGATGTGGAAATCTTTGAGAATAAAACCACGCTGCCGGTTAATGTTGCATTTGAAC
TGTGGGCAAAACGCAATATCAAACCGGTTCCGGAAATCAAAATTCTGAATAATCTGGGCG
TTGATATCGCAGCCAATACCGTTATTTGGGATTATAAACGTGAAGCTCCGGCACATGTTT
CAACCATTGGTGTTTGTACCATGACCGATATTGCAAAGAAACCGACCGAAAGCGCATGTA
GCAGCCTGACCGTTCTGTTTGATGGCCGTGTTGAAGGTCAGGTTGACCTGTTTCGTAAT
GCACGTAATGGTGTTCTGATTACCGAAGGTAGCGTTAAAGGTCTGACCCCGAGCAAAGG
TCCGGCACAGGCAAGCGTGAATGGTGTTACCCTGATTGGTGAAAGCGTTAAAACCCAGT
TCAACTACTTCAAAAAGGTGGACGGTATTATTCAGCAGCTGCCGGAAACCTATTTTACCC
AGAGCCGTGATCTGGAAGATTTTAAACCGCGTAGCCAGATGGAAACCGATTTTCTGGAA
CTGGCAATGGATGAATTTATCCAGCGTTATAAACTGGAAGGCTATGCCTTTGAACATATC
GTGTATGGTGATTTTAGCCATGGTCAGTTAGGTGGTCTGCATCTGATGATTGGTCTGGCA
AAACGTAGCCAGGATAGTCCGCTGAAATTAGAAGATTTCATTCCGATGGATAGCACCGT
GAAGAACTATTTTATCACCGATGCACAGACCGGTAGCAGCAAATGTGTTTGTAGCGTTAT

TGATCTGCTGCTGGATGATTTCGTGGAAATTATCAAAAGCCAGGATCTGAGCGTTATTAG
CAAAGTTGTTAAAGTGACCATCGATTATGCCGAGATTAGCTTTATGCTGTGGTGTAAAGA
TGGCCATGTGGAAACATTTTATCCGAAACTGCAGTAA**AAGCTT**CGGC-’3

MERS Nsp15

5’-
GCCG**GGATCC**GGCCTGGAAAACATTGCCTTTAATGTTGTTAAACAGGGCCATTTTATTGG
CGTTGAAGGTGAACTGCCGGTTGCAGTTGTTAACGATAAAATCTTTACCAAGAGCGGTGT
GAACGATATCTGCATGTTTGAAAACAAAACCACACTGCCGACCAACATTGCATTTGAACT
GTATGCAAAACGTGCAGTTCGTAGCCATCCGGATTTTAAACTGCTGCATAATCTGCAGGC
AGACATCTGCTATAAATTCGTTCTGTGGGATTATGAACGCAGCAACATTTATGGCACCGC
AACCATTGGTGTTTGCAAATATACCGATATTGATGTGAATAGCGCACTGAACATCTGCTTT
GATATTCGTGATAATTGCTCCCTGGAAAAGTTTATGAGCACCCCGAATGCAATCTTTATTA
GCGATCGCAAGATCAAAAAGTACCCGTGTATGGTTGGTCCGGATTATGCCTATTTCAATG
GTGCAATTATTCGCGATAGTGATGTGGTGAAACAGCCGGTTAAATTCTACCTGTATAAAA
AGGTGAACAACGAGTTTATCGATCCGACCGAATGTATTTATACCCAGAGCCGTAGCTGTA
GCGATTTTCTGCCGCTGAGCGATATGGAAAAAGATTTTCTGAGCTTTGATAGCGACGTGT
TCATCAAGAAATATGGCCTTGAAAACTATGCCTTCGAACATGTTGTGTATGGCGATTTTA
GCCATACCACCTTAGGTGGCCTGCATCTGCTGATTGGTCTGTATAAGAAACAGCAAGAG
GGCCATATTATCATGGAAGAAATGCTGAAAGGTAGCAGCACCATCCACAATTATTTCATT
ACCGAAACCAATACCGCAGCCTTTAAAGCAGTTTGTAGCGTGATTGATCTGAAACTGGAT
GATTTTGTGATGATCCTGAAAAGCCAGGATCTGGGTGTTGTTAGCAAAGTTGTTAAAGTT
CCGATCGATCTGACCATGATCGAATTCATGCTGTGGTGTAAAGATGGTCAGGTTCAGAC
CTTTTATCCGCGTCTGCAGTAA**AAGCTT**CGGC-’3

Supplemental Table 2: Primers used for Nsp15 cloning and sequencing.

Name	Sequence
NSP15 Wildtype Primers FWD	5'-ATCATCATCACAGCAGCGGCGTGGATCTTGGTACGGAAAATC-'3
NSP15 Wildtype Primers REV	5'-GCCTCGAGTGCGGCCGCTTACTGCAGCTTGGGATAAAATG-'3
PC013 Vector FWD	5'-TAAGCGGCCGCACTCGAG-'3
PC013 Vector REV	5'-GCCGCTGCTGTGATGATG-'3
Δ0-28 Δ336-347 FWD	5'-TGTACTTTCAATCCAATGCCAACAATACGGTATACACCAAAG-'3
Δ0-28 Δ336-347 Primer REV	5'-ATCCTTGCACCAAAGCATAAAG-'3
NSP15 H250A SDM Primer FWD	5'-GGGTGGCCTTGCTCTTTTAATAGG-'3
NSP15 H250A SDM Primer REV	5'-AGTTGCGAATGAGAAAAATC -'3
NSP15 C291A/C293A SDM Primer FWD	5'-CGCGTCGGTCATCGATCTGTTG-'3
NSP15 C291A/C293A SDM Primer REV	5'-ACCGCCTTCGAAGATCCTGTCTG-'3
NSP15 C291W/C293W SDM Primer FWD	5'-CTGGTCGGTCATCGATCTGTTG-'3
NSP15 C291W/C293W SDM Primer REV	5'-ACCCACTTCGAAGATCCTGTCTG-'3
T7 FWD	5'-TAATACGACTCACTATAGGG-'3
T7 REV	5'-GCTAGTTATTGCTCAGCGG-'3

Supplemental Table 3: RNA substrate sequences for Nsp15 FRET-based Assay.

Name	Sequence	Source (if previously published)
Positive control FAM RNA	5'-FAM-rArArArArArArArG-'3	N/A
Negative control RNA	5'-FAM-rArArArArArArArGrArArArArArA-BHQ1-'3	N/A
RNA1	5'-FAM-rArArArArArArArGrUrArArArArA-BHQ1-'3	[1]
RNA2	5'-FAM-rCrArArCrUrArArArCrGrArArC-BHQ1-'3	[2]
RNA3	5'-FAM-dAdArUdAdA-BHQ1-'3	[3]
Cy5 RNA	5'-Cy5-rCrArArCrUrArArArCrGrArArC-BHQ2-'3	[2]
Positive Control Cy5 RNA	5'-Cy5-rCrArArCrU-'3	N/A

REFERENCES

1 Nedialkova, D. D. *et al.* Biochemical characterization of arterivirus nonstructural protein 11 reveals the nidovirus-wide conservation of a replicative endoribonuclease. *Journal of virology* **83**, 5671 (2009).

2 Guarino, L. A. *et al.* Mutational analysis of the SARS virus Nsp15 endoribonuclease: identification of residues affecting hexamer formation. *Journal of molecular biology* **353**, 1106-1117 (2005).

3 Bhardwaj, K., Sun, J., Holzenburg, A., Guarino, L. A. & Kao, C. C. RNA recognition and cleavage by the SARS coronavirus endoribonuclease. *Journal of molecular biology* **361**, 243-256 (2006).
